# Application of PVD coatings in medical implantology for enhanced performance, biocompatibility, and quality of life

**DOI:** 10.1016/j.heliyon.2024.e35541

**Published:** 2024-08-03

**Authors:** Khondoker Safin Kaosar Saad, Tasfia Saba, Adib Bin Rashid

**Affiliations:** Department of Industrial and Production Engineering, Military Institute of Science and Technology (MIST), Dhaka, 1216, Bangladesh

**Keywords:** PVD coatings, Thin films, Biocompatibility, Corrosion resistance, Osseointegration

## Abstract

Physical vapor deposition (PVD) coating is a versatile and well-liked method for depositing thin films of materials onto surfaces in a range of industries. Due to their numerous functional and aesthetic benefits, PVD coatings are beneficial in several applications, from electronics and optics to automotive and medical equipment. PVD coating technology dramatically improves the effectiveness and quality of medical implants. PVD-coated medical implants improve osseointegration, lower wear and friction, increase corrosion resistance, and have antibacterial properties, which lead to better patient outcomes, fewer complications, and overall higher quality of life for people who need implantable medical devices. The essential concepts of PVD coating and the numerous deposition techniques and materials used are covered at the study's outset. The specific uses of PVD-coated medical implants are then highlighted, including those for orthopedic and dental implants and cardiovascular and neurosurgical devices. The review also emphasizes the critical contribution of PVD coatings to reducing wear and friction, improving corrosion resistance, augmenting biocompatibility, enhancing osseointegration, and aesthetic appeal. The challenges and prospects of PVD coating technologies were further addressed in this article. This review is invaluable for academics, doctors, and businesspeople interested in the beneficial combination of PVD coating and medical implantology.

## 1 Introduction

Biomedical implants are artificial objects or materials placed into a person's body to replace a missing or malfunctioning natural internal part. Orthopedic implants, pacemakers, and artificial heart valves are examples of biomedical implants. Protecting metal implants from the aggressive environment of physiological fluids is essential for their long-term success inside a patient's body [[1], [2]]. However, there are issues with using metallic implants because of poor implant attachment, osteoconductive, corrosion, and wear resistance, which causes wear debris to build and release corrosive ions [3]. An implant device may be impacted by chemical deterioration in two different ways. First, metal ions may create inflammatory reactions in the tissues close to the implant, making the patient uncomfortable and resulting in the device becoming looser. Wear resistance and other mechanical properties must be improved for the metallic implant to work. Second, localized corrosion processes help to generate fatigue fractures, which are the primary cause of mechanical failure of metallic implants due to corrosion fatigue [4]. Physical Vapor Deposition (PVD) can improve material properties and biocompatibility for both cases.

Vaporizing a material in a vacuum chamber and depositing the vapor onto a substrate is known as physical vapor deposition (PVD). PVD can make the deposition of various materials, including metals, ceramics, and polymers. Additionally, it can be utilized on a variety of biomedical implants. The coating material vapor for PVD technology is created via physical processes like evaporation or sputtering. The next step is to coat the object, after which the steam can be applied. PVD technology can now be divided into three broad categories: vacuum evaporation, ion plating, and sputtering plating [5].

The history of Physical Vapor Deposition (PVD) coating traces back to the seventeenth century with early vacuum technology experiments, leading to significant milestones in the nineteenth and twentieth centuries. Sir William Grove's observations in the 1850s on cathode sputtering laid foundational principles for PVD processes, followed by Irving Langmuir's development of evaporation deposition in the 1930s, which marked the technique's initial application. Magnetron sputtering in the 1970s revolutionized PVD technology, enhancing film quality, deposition rates, and compositional control. PVD coatings have since expanded across industries, including automotive, aerospace, electronics, optics, and medical devices. In the medical field, PVD coatings are as critical as implant coatings, offering benefits such as improved biocompatibility, wear resistance, and corrosion protection. Advancements in PVD equipment and processes have further enabled the production of high-performance coatings with enhanced properties, making them increasingly vital for medical implants where durability and reliability are paramount [[6], [7], [8]].

Coatings applied to surgical instruments must be tough, wear-resistant, strong, corrosion-resistant, rigid, and biocompatible. Medical devices with PVD Coating improve performance and visibility. Coatings like TiN and AlTiN reduce galling, enhance edge retention, and speed up tissue release. A surgical instrument coated with TiN or AlTiN is less likely to stick or cling to the surface of tissue it encounters. This helps to facilitate smoother tissue release by lowering the possibility that the instrument may tug or pull tissue when it is being sliced or otherwise worked with [9]. The critical difficulty in ensuring the coatings is biocompatible is necessary for their use in medical equipment. Furthermore, transferring a line-of-sight presents considerable challenges when applying coatings on circular geometries, making it practically unachievable. The temporal span of a triumphant implantation is a pivotal metric for evaluating the efficacy of said implant. However, orthopedic implants face several difficulties and threats that can lead to failure, shortened lifespan, and even risk human life. Aseptic loosening, severe bone loss, fractures, persistent infection, and hypersensitivity reactions to metallic elements are common causes of prosthetic device failure [10]. The potential of coating techniques to enhance the osseointegration of orthopedic implants has been the subject of much research. However, more research is required to confirm their anti-inflammatory potential. Bioactive compound-containing layers can reduce the severity of foreign body reactions [11]. PVD coatings, including their properties, applications, existing issues, and future development prospects, consider other coating materials' performance and performance requirements on medical devices.

This study summarizes the results of 141 relevant studies conducted between 2000 and 2023. Reviews of PVD technology have previously covered a wide range of topics, primarily emphasizing the biocompatibility of medical implants coated with PVD. This review further explains these findings, especially about applications in orthopedics, dentistry, cardiology, and antibacterial functionalization. It specifies the characteristics, uses, difficulties, and the potential of PVD coatings on medical equipment.

## 2 Overview of PVD technologies

**Fig. 1:**
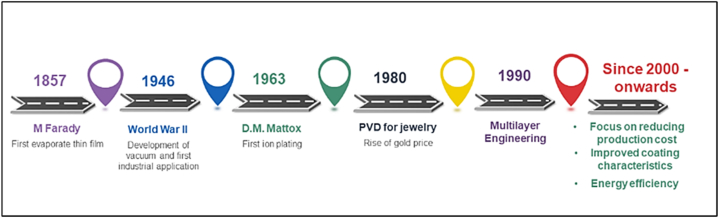
Historical timeline of thin film evolution.

Vacuum technology, electricity, magnetism, and understanding gas chemistry have all contributed to the development of PVD coatings over several centuries. In the past, it needed to be more evident how to manipulate and model materials to fit a specific purpose. In Fig. 1, a historical timeline is drawn for better understanding.

To form thin films on a substrate, several coating methods use physical vapor deposition (PVD), which involves the condensation of evaporated material. Everything operates in a void. There are three techniques: evaporation, ion beam sputtering, and magnetron sputtering. The benefits of each technology vary, and a summary of these differences is provided in Table 1.

**Table 1:**
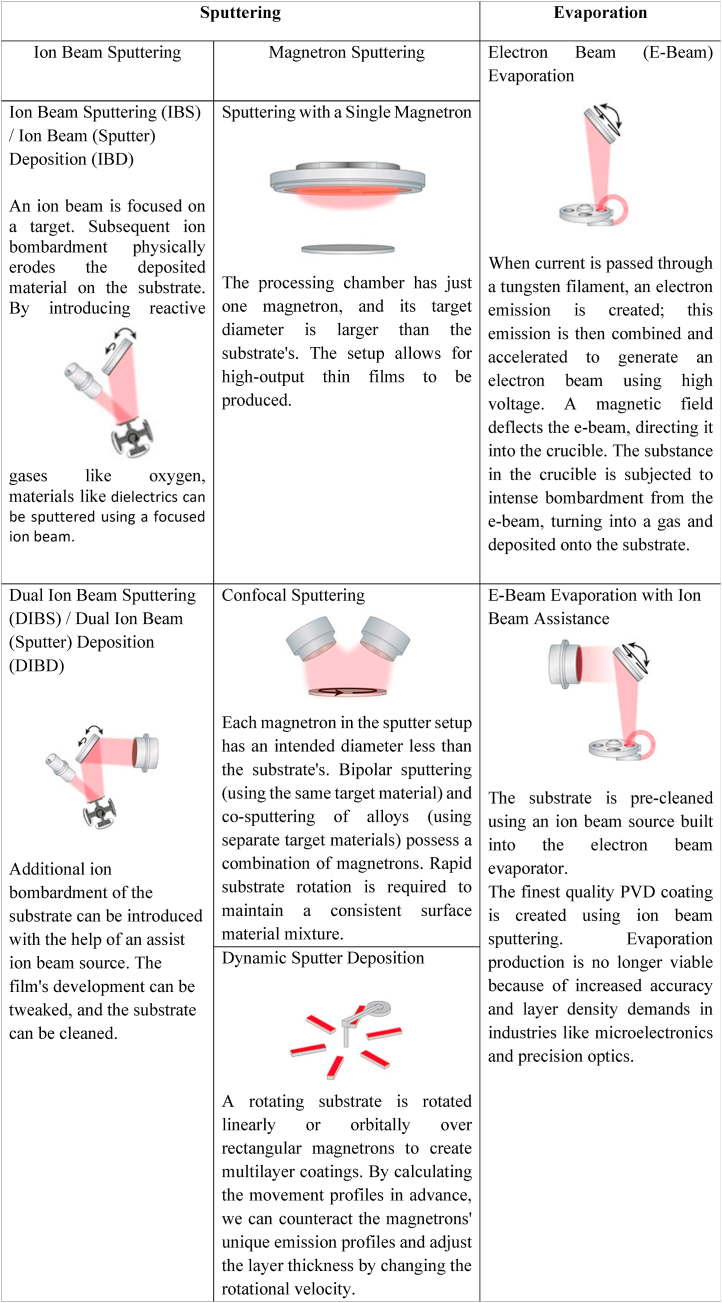
Short note on different technologies of PVD coating.[12]

**Fig. 2:**
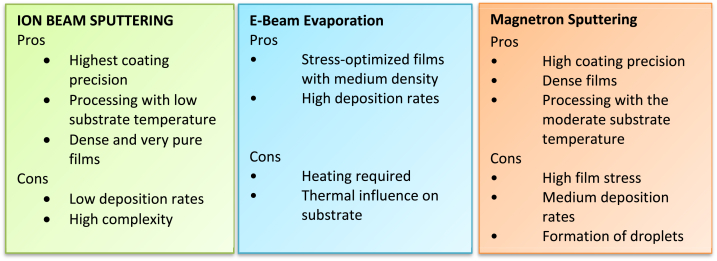
Advantages and disadvantages of the PVD coating techniques [12].

Methods like magnetron and ion beam sputtering are necessary to provide a high yield of working devices. However, these techniques cost more to buy and run and have lower throughput. Beam Evaporation is more economical if a coating procedure for high-temperature materials with high deposition rates is required. The advantages and disadvantages of the PVD coating techniques have been addressed in Fig. 2.

## 3 PVD coating methods/techniques

Physical vapor deposition, or PVD, is a standard method for depositing thin films in various settings. It can be used for multiple purposes, including improving tribological behavior, increasing optical qualities, revamping aesthetics, and more [13]. Sputter systems come in various types, including ion beam and magnetron sputtering [14].

**Fig. 3:**
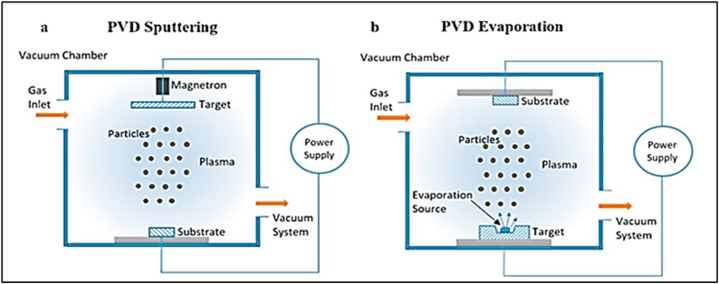
Schematic diagram of two physical vapor deposition (PVD) techniques, namely (a) sputtering and (b) evaporation coating processes [14].

The coating material is deposited onto a substrate by directing an ion-electron beam onto a target. In magnetron sputtering, a plasma-based coating process, negatively charged source materials are smashed by positively charged particles from a magnetically confined plasma. When two objects collide, the atoms in each are pushed apart and eventually settle on the substrate shown in Fig. 3(a–b).

An ion-electron beam must be directed at a target to sputter material onto a substrate. Before applying the coating, the region is typically placed in a vacuum chamber with an inert gas. After being negatively charged, the source material acts as a cathode and gives off electrons. The free electrons interact with the negatively charged electrons surrounding the gas atoms. When the electrons in a gas are ejected, the resulting ions have a lot of energy and are positively charged. These ions are then attracted to the source material, colliding at such high velocities that they break apart particles as small as atoms [14].

However, in conventional e-beam evaporation (Fig. 3(b)), the target acts as the cathode, and the water vapor is incorporated into the evaporation source. The particles of atomic size can fade when subjected to enough heat from an electron beam. The gas molecules in the reactor will clash with the propelled particles, creating plasma and increasing their velocity. After that, the plasma moves through the deposition chamber, becoming most concentrated in the reactor chamber's sweet spot. Particle deposition into a substrate allows for the progressive creation of compacted layers, essential for successfully constructing a high-quality film on a substrate [15].

**Fig. 4:**
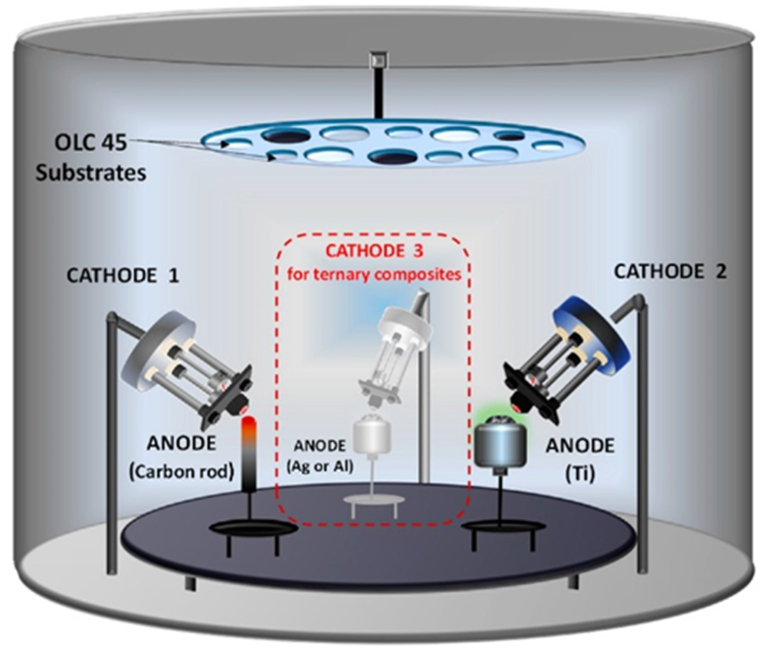
The schematic view of the experimental set-up [17].

Since sputtering permits deposition at temperatures as low as 50 °C, it reduces the substrate's environmental impact [16]. However, the evaporation method has the drawback of infecting the film during the dispersion of the evaporated source [14]. It is restricted to the materials that may be deposited due to their melting temperature. The cost and effort involved in sputtering are more significant than other methods, but the resulting film is of higher quality and more uniform. In addition, sputter deposition is highly complex. With this technique, deposition of a wider variety of materials is possible and more precise regulation of the composition of the layered deposition films could be maintained. Fig. 4 shows the components of a PVD reactor, including the vacuum chamber, high-voltage energy supply, and electrodes [14].

By comparison, evaporation shines in settings calling for both high throughput and volume output, but it cannot be easily scaled. Selecting the best approach balances yield, cost, film quality, and throughput. The method's commercial viability and customers tastes are equally important factors. Table 2 compares the two approaches based on the various process parameters.

**Table 2:** Comparing process parameters between sputtering and evaporation [14].

Process Parameter	Evaporation	Sputtering
Absorption	Less absorbed gas without film	Higher absorption
Vacuum	High	Low
Adhesion	Low	High
Uniform film	Less	More
Deposition rate	It can be very high	Low except for pure metals and dual magnetron
Grain size	Bigger	Smaller
Atomized particles	Highly directional	More dispersed
Deposited species energy	Low (∼0.1–0.5 eV)	Can be high (1–100 eV)

### 3.1 Substrates

PVD coatings can be applied to various substrates, including steel, aluminum, copper, titanium alloys, and polymers (such as polycarbonate). Because the microstructure profoundly affects the coating's corrosion resistance, PVD coatings are best applied to corrosion-resistant substrates. The unique columnar structure of PVD films results from the line-of-sight approach, often coupled with a relatively insignificant proportion of porosity. There is a chance that substrate degradation processes can enter the coating through its pores [18]. This is a crucial consideration when selecting a substrate for production. For example, corrosion-resistant substrates are favored because the microstructure greatly influences the corrosion performance of PVD coatings. Lead-free brasses with bismuth and silicon content are also machine-friendly and suitable as decorative substrate materials. Substrate compatibility with PVD procedures should be considered throughout the selection process. Raw material cost, usability, and the possibility of mass production are significant factors that influence the industry [19].

### 3.2 Coating material

Titanium-based material like TiN, is a widely used PVD coating, excellent at avoiding wear. Many different materials are also there which could be deposited as single or multiple-layer coatings, including aluminides, MCrAlYs, Al_2_O_3_, ZrO_2_, ZrN, CrN, TiCN, TiAlN, and diamond-like coatings (DLCs).

#### 3.2.1 Titanium-based coating

Titanium is not widely used in engineering because of its low hardness, unimpressive antifriction qualities, and significant susceptibility to wear. It is commonly used to improve the stiffness of metal matrix composites. Impressive new materials will be created when titanium is paired with other metals. Based on this premise [20], investigated binary (Ti–C or Ti–Ag) and ternary (Ti–C, Ag, and Al) Ti-containing thin films. Glass, silicon wafers, and OLC 45 can all be used to grow Ti (99.99 % purity), Ag (99.9 % purity), and Al (99.99 % purity) based on ternary composite films. The schematic view of the experimental set-up is depicted in Fig. 4.

Table 3 lists the critical operational factors that affect the deposition process, such as the deposition rate, the ultimate thickness for each substance that evaporates, and the average pressure throughout the coating. If the heating filament's current intensity is high, U_a_ is the voltage placed across the electrodes, and I_a_ is the strength of the arc current.

**Table 3:** The parameters for ternary and binary thin film synthesis [20].

Parameters	Ternary						Binary	
	Ti–C–Ag			Ti–C–Al			Ti–C	Ti–Ag
	C	Ti	Ag	C	Ti	Ag		
I_a_ (A)	1.00	1.12	0.30	1.0	1.12	0.5	0.3	0.7
I_f_ (A)	76	38	36	76	38	38	57	54
U_a_ (KV)	2.0	0.8	1.1	2.0	0.8	1.3	1.35	0.8
Thickness (nm)		400			1800		125	100
Rate of Deposition(Å/s)		0.76			0.86		0.06	0.05
Pressure During Deposition (Pa)		2.6 × 10^−3^			9.9 × 10^−4^		7.0 × 10^−4^	6.0 × 10^−4^

#### 3.2.2 Binary coating of titanium

The distinctive visual appeal of TiN coatings has piqued the interest of many, leading to investigations into whether they can increase the wear resistance and longevity of instruments. Storz [21] examined uncoated and coated scissors made from 420 stainless steel and found no discernible difference in performance. The results demonstrated that the blades of the uncoated scissors required regrinding after an average of 12,000 uses. However, the coated pair of scissors could be used around 100,000 times before they needed to be replaced. The extreme improvement resulted in an eight-hundred-percent increase in durability [22].

Corrosion and wear parameters of 316L stainless steel and UHMWPE connectors were studied in horse serum after being coated with DLC, TiN, or Micronite through magnetron sputtering. Micronite is a novel coating that is applied using physical vapor deposition technology. It is paired with a low-friction coating material to enhance its tribological capabilities. The “micronite” method results in a much lower coefficient of friction for TiN-coating. The testing revealed that the DLC coating did not lessen friction or wear. Hosseini et al. [23] found that using a TiN coating lowered the friction coefficient by a factor of four while using Micronite reduced it by five.

Sonoda et al. [24] employed a DC magnetron sputtering technique to deposit pure titanium (Ti) and titanium oxide (Ti–O) on the surface of a titanium alloy. According to the findings, a Ti–O coating can increase titanium alloys' surface hardness and wear resistance. They discovered that the ductility of the titanium alloy was improved by coating it with pure titanium (Ti) and heating it to 800 °C. Pappas and Buechel tested the effects of cyclic vibration in deionized water at 37 °C and a load of 02200N on a Ti–6Al–4V artificial femur coated with TiN. A titanium nitride (TiN) layer 9 μm thick was deposited using ion plating on the substrate [25].

**Fig. 5:**
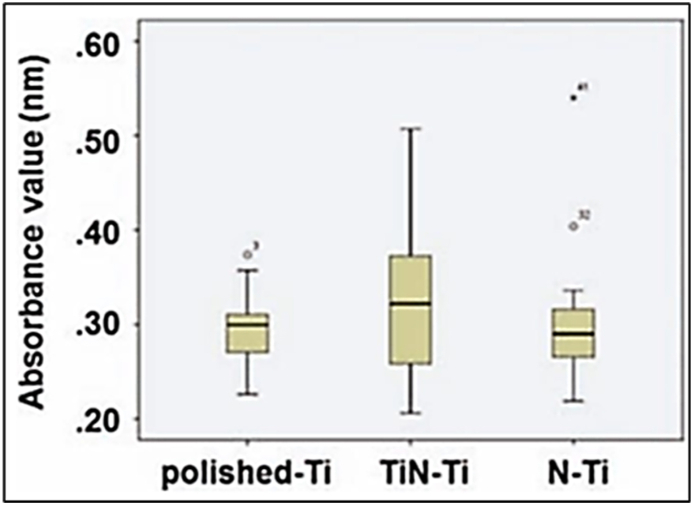
Absorbance values of Streptococcus mutans across several experimental groups. Circles are used to represent outliers [27].

The joints were polished to a surface finish of 0.04 μm Ra before and after applying the coating. CoCrMo has better wear resistance than Ti–6Al–4V. Surface characteristics of Grade IV titanium were modified using DC magnetron sputtering and plasma nitriding by Ji et al. [26]. Afterward, the response of Streptococcus mutants to three different types of titanium was measured using a crystal violet staining evaluation (Fig. 5): (a) polished titanium (the control group), (b) titanium changed using DC magnetron sputtering (group TiNTi), and (c) titanium subjected to plasma nitriding (group N–Ti). When tested under the same conditions, the S. mutant's adhesion to the control, TiN–Ti, and N–Ti surfaces did not differ substantially (P > 0.05) [27].

**Fig. 6:**
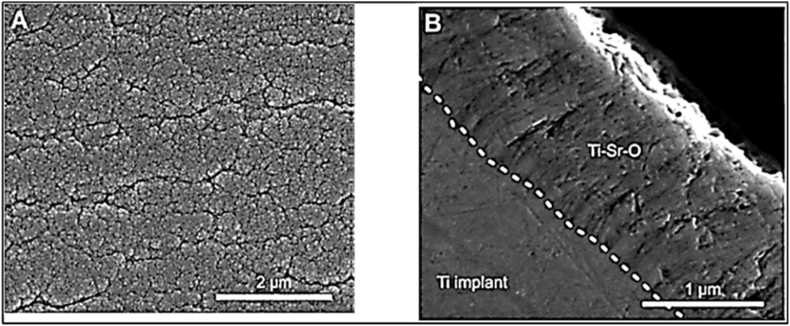
SEM images of Ti–Sr–O: (a) surface and (b) cross-section [28].

Vincent et al. [28] have developed a PVD magnetron sputtering procedure to create a Ti–Sr–O functionalized coating in contrast to the clinically acceptable and well-established surface known as SLActive. Strontium is slowly released from this covering. Nanopatterned Ti–Sr–O on titanium surfaces showed continuous strontium release, as shown in the results (Fig. 6(a and b)).

#### 3.2.3 Ternary coating of titanium

Because of their extreme hardness, ternary coatings like TiN and CrN can increase the durability of surgical tools. However, these items still have a high coefficient of friction and are not resistant to chemical damage, even after several washings and disinfection cycles. While adding Al can improve the coating's resistance to wear and corrosion, adding C can reduce the coating's friction coefficient. Due to its excellent chemical barrier capabilities, low reflectivity, good adhesive characteristics, low susceptibility to wear, and outstanding abrasion resistance, the AlTiN coating is frequently used on surgical equipment [5].

In another work, Noori et al. [29] applied the CAE-PVD approach to deposit CrN/TiN and CrN/ZrN nanostructured multilayer coatings on a Ti–6Al–4V alloy substrate. The results showed that the coated samples with the TiN top layer had more microparticles than those with the other two coatings because of their increased roughness. Additionally, their increased contact angles were noted. It was proposed that more excellent air trapping on their surface is the cause of this phenomenon. The microhardness test findings indicated that two-layered coatings had a higher hardness than single layers. The results of the scratch tests showed that the critical load values for the nanostructured multilayer coatings of CrN/TiN and CrN/ZrN were high. Because of its unique design, smoother surface, and denser structure than any of the evaluated samples, the CrN/ZrN multilayer coating demonstrated the best corrosion resistance, according to corrosion assessments.

#### 3.2.4 TiCN-based coating

Because of their favorable mechanical properties and straightforward, cost-effective production, austenitic stainless steels find extensive use in orthopedic applications [30]. A study found that pitting or degradation frequently occurred in the crevices where stainless-steel implants had been inserted after removing them [31]. An implant could fail if exposed to both pollution and fatigue. PVD hard ceramic coatings are commonly used to remedy the situation. Titanium carbonitride (TiCN) is a challenging and conductive material with excellent electrical and thermal properties. Since this is the case, TiCN films are used as a coating for tool steels and semiconductors [32]. Corrosion current density in TiCN-coated AISI 304 stainless steel was reduced by as much as three orders of magnitude, according to research by Feng et al. [32] Its non-cytotoxic nature and mechanical and corrosion properties make it appealing for biomedical applications. Despite the proper biological response, consistent performance in real-world implant devices requires further improvement of the PVD layer's electrochemical behavior [33].

#### 3.2.5 Hydroxyapatite (HAP) based coating

Hydroxyapatite, a calcium phosphate mineral (Ca_10_(PO_4_)6(OH)_2_), is notable in the science of geology. By including oxides in the hydroxyapatite (HAP) coating, Zhao et al. [34] were able to boost the film's adhesion properties. Using RF magnetron sputtering, researchers successfully produced a bio-composite coating of HA(+ZrO_2_+Y_2_O_3_) on Ti–6Al–4V. The study synthesized hydroxyapatite (HA) powders via a sol-gel method, starting with (CH_3_O)3PO_4_ and Ca (NO_3_)_2_·4H_2_O, followed by drying, calcination at 800 °C, and ball milling. Characterization confirmed high-purity, polycrystalline HA powders with a spherical morphology of sub-micrometer to nanometer sizes. To create HA(+ZrO_2_+Y_2_O_3_) sputtering targets, HA powders were mixed with yttria-stabilized zirconia, ball-milled, and cold-pressed into composite targets. XRD analysis verified the composition. Ti–6Al–4V plates were used as substrates for coatings after mechanical polishing, etching in 30 % HNO_3_, ultrasonic cleaning with acetone and ethanol, and rinsing with de-ionized water.

This approach demonstrates practical synthesis and preparation methods for HAP-based materials used in biomedical applications. The covering was bolstered with apatite, a mineral whose chemical composition is identical to the inorganic phase of natural bone (CO_3_^2−^). This finding demonstrates the coating's high biocompatibility and positive biological activity. Delamination at the composite coating/matrix interface was not seen after prolonged immersion in the simulated physiological fluid. That means the composite coating is still remarkably durable [5].

#### 3.2.6 Ta coating

The transition metal tantalum and its various oxides, nitrides, and other derivatives have been proven to promote cell adhesion, growth, and differentiation. To alter the chemical makeup of a titanium alloy, I first coated it with TaC and then created special TaCN and TaN coatings. These coatings can increase cellular growth and development on the surface by encouraging early cell attachment. Coatings of TaCN and TaN increase cell proliferation rates to a more significant extent [35].

#### 3.2.7 DLC coating

Diamond-like carbon (DLC) coatings are highly valued in biomedical applications for their excellent mechanical properties, low friction coefficient, and biocompatibility [36]. They exhibit bio-inertness and non-toxicity towards osteoblast-like cells in simulated body fluid [[37], [38], [39]], making them ideal for orthopedic, dental, and cardiovascular applications. DLC coatings improve endothelization on medical implants and reduce thrombotic risks [40], with ongoing efforts to enhance hydrophobicity and wear resistance through doping with elements like fluorine (F) [41].

Hydrophobic DLC coatings inhibit blood cell adsorption, enhancing their anti-thrombogenic properties [42,43]. Fluorine-modified DLC surfaces show significantly improved anticoagulant effects, reducing platelet adhesion and activation [42]. Similarly, silicon-doped DLC (Si-DLC) coatings improve frictional properties in orthodontic applications [44]. DLC coatings doped with chromium, titanium, copper, and silver enhance wear resistance and antibacterial characteristics [[43], [44], [45], [46]].

**Fig. 7:**
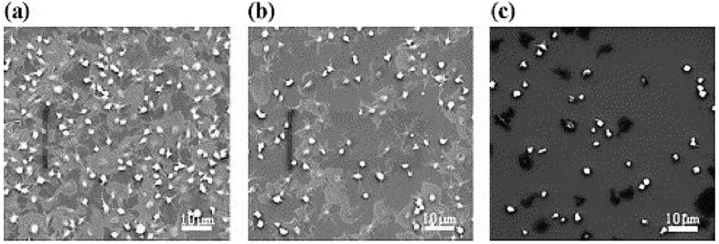
Morphology of adherent platelets on (a) Si, (b) diamond-like carbon, and (c) F-doped diamond-like carbon surfaces (60 min incubation in platelet-rich plasma) observed using SEM [55].

In joint arthroplasty, DLC-coated articulating surfaces enhance bio tribological behavior and longevity, promoting better bone-implant integration and reducing infection risks [[47], [48], [49]]. DLC coatings also mitigate thrombosis risks on stents, making them promising for vascular interventions [50], particularly when modified with fluorine to improve surface chemistry and wettability [51]. Research explores novel DLC modifications such as zinc-doped DLC (Zn-DLC), which enhances osteogenesis and bone fracture healing [52,53]. DLC coatings on artificial heart valves aim to improve operational lifespan by enhancing endothelial cell adhesion and growth [54]. Fig. 7(a–c) presents adherent platelets on Si, DLC, and F-doped DLC surfaces, which is observed using Scanning Electron Microscope (SEM).

In summary, DLC coatings are versatile biomaterials with significant potential across various medical fields. Ongoing research to optimize their properties and biocompatibility profiles is crucial for advancing their clinical applications.

#### 3.2.8 Ag-based coating

**Fig. 8:**
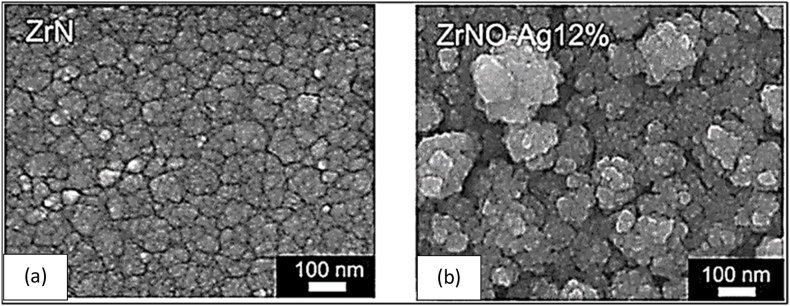
Surface morphologies of (a) ZrN (top) and (b) ZrNO–Ag12 % (bottom) coatings are shown (SEM micrographs) [57].

Metals such as silver or copper have been employed to achieve antibacterial functionalization of materials intended for medical applications [56]. Fig. 8 shows that Chang et al. employed reactive magnetron sputtering to create ZrN, ZrO_2_–Ag, and ZrNO–Ag coatings with varying amounts of Ag. The ZrNO–Ag material was found to have a porous structure with monoclinic ZrO_2_, fcc Ag, and Zr_2_ON_2_ phases, according to research by Chang et al. [57]. This research set out to examine the effect of ZrO_2_–Ag and ZrNO–Ag coatings on the proliferation of human gingival fibroblast (HGF) cells and the antibacterial efficacy of these coatings against *Actinobacillus actinomycetemcomitans (A. actinomycetemcomitans)* and *Staphylococcus aureus (S. aureus).* Compared to the uncoated model, HGF cell viability and proliferation were improved on titanium samples coated with ZrNO–Ag2%, ZrO_2_–Ag5%, ZrN, and ZrNO–Ag12 %. The most efficient concentration of porous ZrNO–Ag12 % coating (11.8 at. %) showed rapid antibacterial effects and satisfied the HGF's cell survival requirement (Fig. 8(a and b)).

**Fig. 9:**
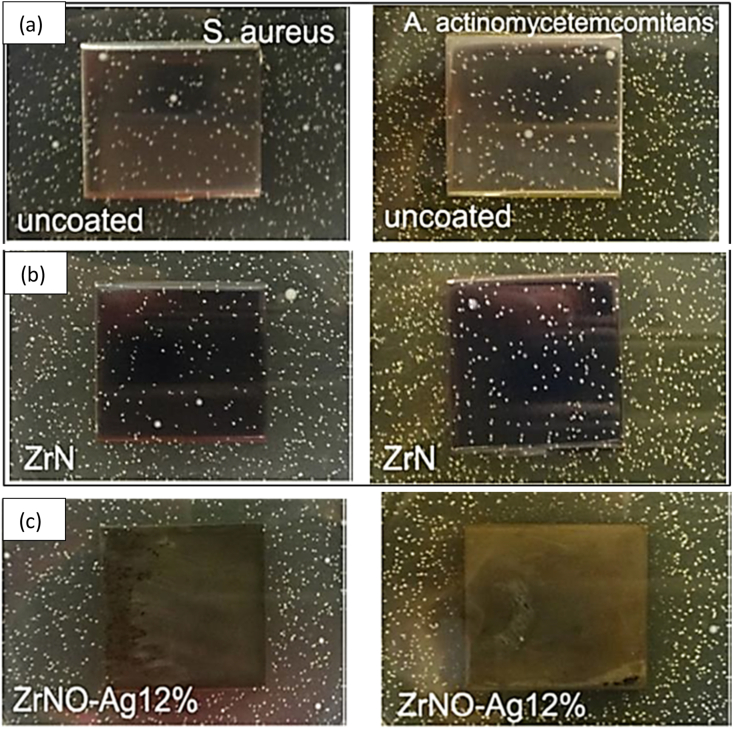
Bacterial viability test results on (a) uncoated, (b) ZrN, and (c) ZrNO–Ag12 % coated Ti sample plates[57].

Colonies on the agar plates grew *A. actinomycetemcomitans* and *S. aureus*. Each sample indicated that the growth rate of *A. actinomycetemcomitans* on Ti was 46 cfu/cm^2^, whereas on ZrN, it was 47 cfu/cm^2^, and on ZrNO–Ag12 %, it was 0.5 cfu/cm^2^. The average number of *S. aureus* cfu/cm^2^ on Ti, ZrN, and ZrNO–Ag12 % was 36, 38, and 1, respectively (Fig. 9(a–c)) [57].

Dan et al. [21] examined the fundamental characteristics of coatings made on NiTi alloy surfaces using magnetron sputtering (Table 4). They discovered that the DLC coating material had a reduced coefficient of friction and that the coatings may successfully stop Ni ions from precipitating.

**Table 4:** Basic properties of surface coating on NiTi alloy substrate prepared by magnetron sputtering [21].

Coating	Thickness (μm)	Hardness (GPa)	Membrane Binding	Coefficient of friction
Ti/DLC		18.8(VH)	37.5	
DLC	0.253–1.88		60	0.07
Ti/TiN		24.0(VH)	82.3	
Ti/CN		23.1(VH)	63.3	
CN/SiC		5.23(NH)		0.173
Zr	3.5			
Zr–Ti	4.0			
ZrN	0.66	19.5(NH)		0.3
Polyamide	0.7–0.9			
Calcium phosphate			0.1–0.5	

#### 3.2.9 The metal-organic composite coating on magnesium alloy implants

**Fig. 10:**
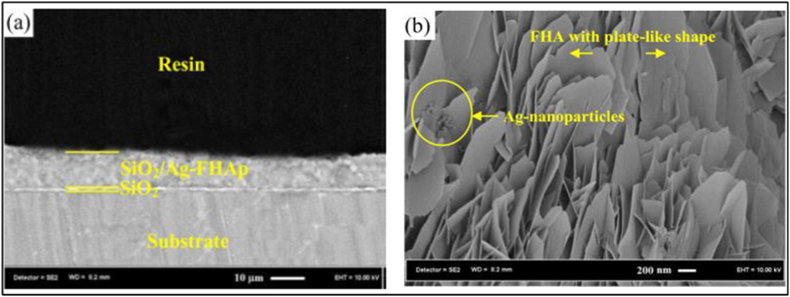
Cross-sectional SEM of SiO_2_/Ag-FHA coated Mg alloy specimens' resin (a); SEM of overlayer surface [58].

A novel bi-layered nanostructured coating of silica (SiO_2_) and silver-doped fluorohydroxyapatite (Ag-FHAp) was successfully fabricated by Bakhsheshi-Rad et al. [58] on a biodegradable Mg-1.2Ca-4.5Zn alloy using PVD and ED techniques. Fig. 10 (a) shows that the nano-SiO_2_ substrate had a 1 μm thickness and a closely packed columnar microstructure. The Ag-FHAp overlayer, on the other hand, was roughly 10 μm thick and was made up of giant plate-like crystals and minute spherical particles. Coating the Mg alloy with a double layer of SiO_2_/Ag-FHAp makes it more corrosion-resistant than uncoated samples (Fig. 10 (b)). Because the surface wettability of magnesium alloy is significantly improved by putting an Ag-FHAp coating over nano-SiO_2_ layers, it has been recognized as a potential material for implant applications. This enhanced wettability facilitates cell attachment to the implant surface.

#### 3.2.10 Metal oxide coating

Hübsch et al. [59] investigated the feasibility of using transparent PVD coatings on zirconia ceramics to prevent the material from degrading at low temperatures, making it suitable for the dentistry industry. Regular 3Y-TZP discs were coated with titanium oxide (TixOy) and titanium oxide-alumina-titanium oxide (TixOy-AlxOy-TixOy) multilayers, each with a thickness of around 50 nm. Hübsch et al. [59] found that applying PVD coatings effectively inhibited the transformation of zirconia ceramic from its tetragonal phase to the monoclinic phase. The effectiveness of the TAT (TixOy-AlxOy-TixOy) coating surpasses that of the Ti coating. According to the authors' idea, some vertical diffusion channels along the edges of the columnar titanium oxide grains may have been interrupted due to the existence of horizontally overlapping grain boundaries in the intervening alumina layer. In the long term, it won't be able to prevent the zirconia surface from undergoing the t-m phase change. PVD coatings come in two varieties, both of which are suitable for usage in dental offices.

#### 3.2.11 Other coatings

Alias et al. [60] employed physical vapor deposition (PVD) magnetron sputtering to fabricate a thin Ag/AgTa_2_O_5_ nanocomposite film. In this research, preventing microorganisms from sticking to surgical tools was the top priority. The mechanical properties of the film may be improved through heat treatment. The adhesion strength increased by almost 152 % after being subjected to an annealing process at 400 °C, reaching 2916 ± 147. The amorphous structure's tighter connection to the 316L stainless steel substrate is responsible for the improvement. In Table 5, the overall summarized information is presented to provide an idea of some parameters (hardness, friction, thickness, color) and properties of PVD coating. At the same time, different kinds of material combinations are used as substrates. This table will help you use suitable coating material for biomedical implants.

**Table 5:** Concise description of parameters and properties of different chemical deposition of coatings

Chemical composition	Color	Degradation temperature (°C)	Hardness (HV)	Friction co-efficient*	Thickness (μm)	Properties
TiN based	Golden yellow	600	2600	0,40	2–5	All mechanical and chemical attributes are medium, and their utility and applicability are general.
TiCN based	Violet-Grey	400	3800	0,25	1–4	Features high hardness, low friction coefficient, and moderate temperature resistance.
AlTiN based	Black, Purple	900	3600	0,55	1–4	Super-hard, heat-resistant, and resistant to oxidation
CrN based	Silver Grey	700	2400	0,35	2–10	Thin-applied coatings provide a low friction coefficient, excellent surface quality, and high coating tenacity.
AlCrN based	Fume Grey	1000	3600	0,50	1–7	Extremely durable, resistant to heat and oxidation, and well-suited for dry chip removal when cooling is inadequate.
TiSiN based	Bronze	1100	4200	0,50	1–4	The nano-composite structure, high heat resistance, great oxidation resistance, and excellent hardness make this material ideal for chip removal and the dry cutting of rigid materials.
TiCrN based	Golden yellow	600	3000	0,30	1–7	The body comprises low-friction intermediate phases in a low-friction inter-ceramic bind structure.
ZrN based	Bright yellow	450	3000	0,30	1–5	Highly abrasion-, adhesion-, and winding-resistant surfaces with excellent surface characteristics.
AlTiCrN based	Black Purple	950	3700	0,50	2–10	Extremely high thermal hardness, toughness, resilience to high temperatures, and low friction coefficient
AlCrTiN based	Blue-Grey	900	3700	0,30	2–10	Surface characteristics are enhanced, and friction is reduced in the Lubrica Tribo PVD coating version of the Lubrica Top layer.
TiN based	Golden yellow	600	2600	0,40	0,5–2	Extremely high thermal hardness, toughness, resilience to high temperatures, and low friction coefficient

## 4 Property enhancement

In addition to biocompatibility considerations, surgical tools possess distinct criteria for attributes such as strength, hardness, stiffness, toughness, wear resistance, and corrosion resistance.

### 4.1 Wear resistance of biomedical materials

The term “wear” refers to a variety of surface damage-related events. According to the conventional definition, it is “damage to a solid surface, generally involving progressive loss of material, due to relative motion between that surface and a contacting substance or substances.” Plastic deformation at a material's surface can cause wear damage. Any surface deterioration, such as material removal, displacement caused by plastic deformation, topological changes (such as fracture), and surface chemical changes (such as oxidation), can result in wear damage to a material's surface [61].

The coatings applied on surgical tools are required to exhibit resistance against abrasion, bacterial proliferation, cleaning agents, and little light reflectance. The Titanium Nitride (TiN) coating stands out as the most frequently preferred option among the four physical vapor deposition (PVD) coatings offered by Surface Solutions Company to improve the performance of traction, drilling, needles, and other components requiring resistance against wear. The aesthetic quality of the TiN coatings is what makes them stand out. Storz [25] compared uncoated and coated 420 stainless steel scissors to determine the possible influence of TiN coating on the improvement of wear resistance and extension of an instrument's operating lifespan. The CrN coating, which the Star Arc firm commonly applies, exhibits notable attributes such as exceptional toughness, resistance to wear, and protection against corrosion. This coating is extensively used to manufacture surgical instruments, particularly balls, centers, and knives.

**Fig. 11:**
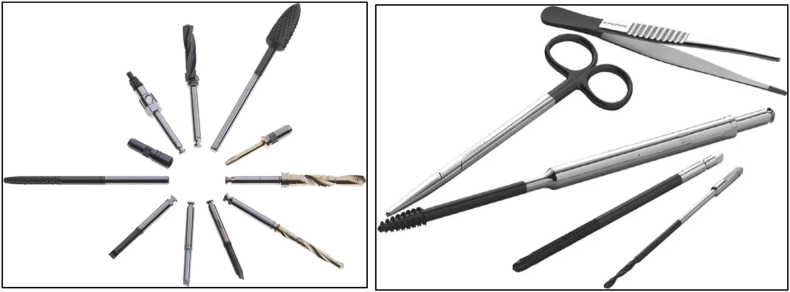
PVD-coated surgical instruments [63].

Based on the research results [62], it has been determined that scissors with a coating have a much longer lifespan compared to those without a layer, with an increase of 800 %. The coated scissors are also exceptionally long-lasting; they can make around 100,000 cuts before the blades need to be sharpened again. Compared to the status quo where scissors are untreated, this represents a dramatic increase in quality of life of around 12 thousand percent. Fig. 11 represents some of the PVD-coated surgical instruments used for surgeries [63].

Moreover, implementing AlTiN coating guarantees an extended operational lifespan for the surgical instrument when subjected to demanding surgical conditions. Utilizing AlTiN coatings from the ion bond corporation presents a viable alternative to the brutal chromium treatment of surgical and dental medical equipment. This substitution is justified by the superior characteristics of TiAlN coatings, including improved adhesion, enhanced resistance to wear, and outstanding performance in washing and disinfection processes involving acids and alkalis [25]. CoCrMo material has better wear resistance than Ti–6Al–4V. After putting a Ti–6Al–4V femoral joint through 10 million cycles, the data showed that the TiN coating was holding up well. The Ionbond company manufactures coatings used on a variety of spinal implants. Crucial components such as spinal discs, pedicle screws, and spine guide rods can significantly improve titanium alloys' wear resistance.

Another important and valuable alloy can be NiTi alloy for medical implants. Still, the primary challenge encountered in using NiTi alloy implants within the medical field pertains to the proclivity of their surfaces to induce thrombosis. To investigate the corrosion and wear characteristics of 316L stainless steel and UHMWPE, Hoseini et al. [64] employed magnetron sputtering to fabricate DLC, TiN, or Micronite coatings on 316L stainless steel.

Previous studies have provided evidence that employing post-polishing procedures on cathodically deposited TiN coatings can effectively enhance wear resistance and minimize the duration of the running-in period. Panjan et al. [40] described manufacturing technology combining hybrid mixed vacuum arc and magnetron sputtering as noteworthy and captivating. The invention of this material was motivated primarily by a desire to improve the crystalline lattice's mechanical strength and wear resistance in wet environments [65,66].

In their study, Wang et al. looked at the effects of varying the reactive nitrogen (N_2_) partial pressure on cathodically produced TiBN/TiAlSiN nano-multilayered coatings. Hardness (34 GPa), H/E, and H3/E*2 increased when the layer was deposited at a partial pressure of 2.0 Pa, and the coating also displayed a defect-free, smoother surface and a dense microstructure. It possessed a low friction coefficient (0.28) and a strong wear resistance (4.3107 mm^3^/Nm) [67].

**Fig. 12:**
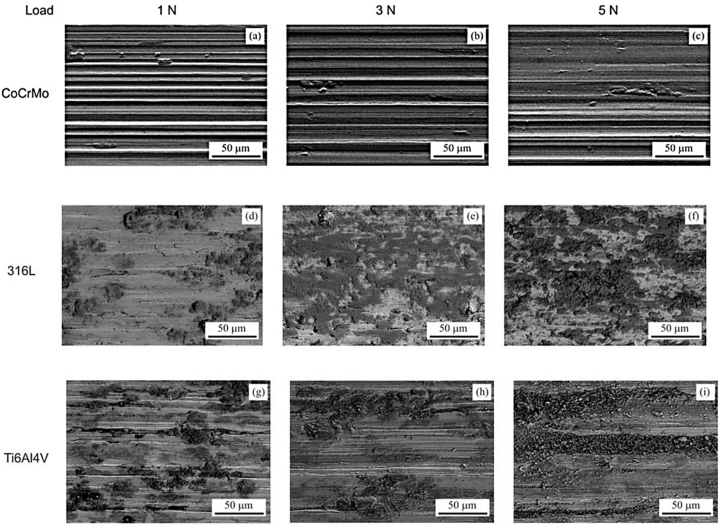
SEM micrographs of the wear tracks formed on the surfaces of (a–c) CoCrMo, (d–f) 316 L, and (g–i) Ti–6Al–4V samples [68].

Fig. 12(a–c) shows how the CoCrMo alloy's contact surface topography exhibited abrasive wear characteristics due to numerous minor grooves aligned parallel to the sliding direction. The 316 L surface topographies worn over time can be visualized in Fig. 12(d–f) as relatively smooth, brilliantly colored regions with dark-hued oxide islands. The Ti–6Al–4V alloy showed the lowest wear resistance, and its samples had the most ragged surface topography, including plastic deformation and grooves parallel to the sliding direction (Fig. 12(g–i)) [68].

Cao et al. [69] noticed a change in the favored crystallographic orientation of TiAlN coatings produced using FCVA when the bias voltage was increased from 50 V to 75 V. Specifically, the favored direction shifted from the (200) plane to the (111) plane due to the increase of atomic mobility and lattice distortion. The observed phenomena of increased hardness (measured at 30.3 GPa) and enhanced wear resistance (estimated at 4.4 x 10^5 mm^3/Nm) can be attributed to heightened atomic mobility and lattice distortion. In contrast to coatings deposited at 0 V, the arc-generated TiNiN coatings by Akhter et al. exhibited a notable enhancement of 85 % in terms of wear resistance [70].

The study conducted by Lin et al., observed a decrease in residual stress from 5.67 to 3.75 GPa, accompanied by a 16 % increase in wear resistance when the thickness of the Ti interlayer in TiZrN coatings increased from 50 to 250 nm [71].

**Fig. 13:**
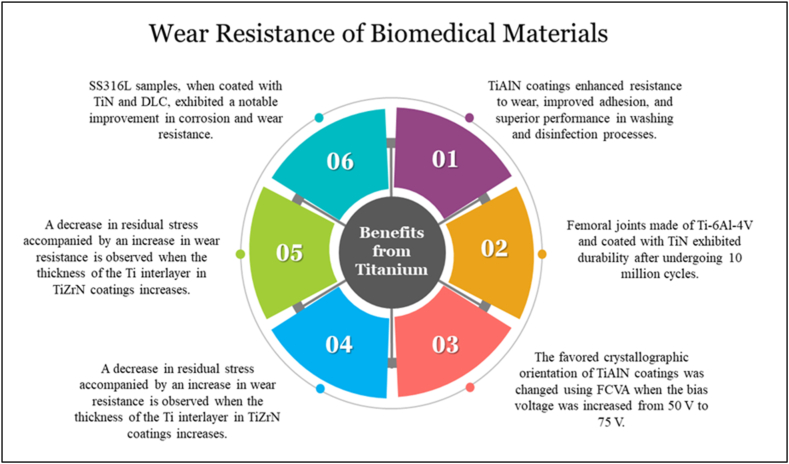
Summary of benefits of Titanium-based coatings in various aspects.

Chang et al. developed a novel multilayer coating consisting of AlTiN, CrN, and ZrN utilizing a cathodic arc technique for tribological applications, as evidenced by the cross-sectional transmission electron microscopy (TEM) micrograph. The researchers observed that implementing an AlTiN/CrN/ZrN multilayer structure significantly reduced residual stress levels, thus enhancing resistance [72]. Fig. 13 demonstrates a summarized description of the usage of Titanium as a tool of wear resistance.

By carefully adding silicon (Si) and boron (B) to titanium aluminum nitride (TiAlN) and chromium aluminum nitride (CrAlN) coatings, a lubricious layer of silicon dioxide (SiO_2_) and boron oxide (B_2_O_3_) or boric acid (B(OH)_3_) can form at temperatures between 800 and 900 °C. This coating reduces friction and increases durability significantly. A lubricating SiO_2_ and B_2_O_3_/B(OH)_3_ layer is produced by adding Si and B to the TiAlN and CrAlN coatings. Within the 800–900 °C temperature range, this layer significantly lowers friction and improves wear resistance. When a tribological system contains soft metals like silver (Ag) and copper (Cu), a tribofilm is formed with a high concentration of these elements. At temperatures up to 500 °C, this triboflim shows its best qualities: low friction and high wear resistance [73].

### 4.2 Corrosion resistance in biomedical implants

Materials for biomedical implants must meet stringent criteria, including high corrosion resistance. The human body is a potentially corrosive environment for biomedical implants due to biological fluids, salts, enzymes, and pH fluctuations. When natural tissues, bones, or organs become damaged or sick, biomedical implants are often implanted into the human body as replacements. Metals, polymers, ceramics, and composites are just some materials that can be used to create these implants. Corrosion resistance is especially crucial for metal implants, such as those composed of stainless steel, titanium, cobalt-chromium alloys, and other materials, because of the hostile physiological environment in which they are placed. These implants must be long-lasting, suitable for human use, and resistant to corrosion and other environmental hazards. Damaged tissues, inflammation, and other difficulties can result from corrosion's release of toxic ions or particles into the body.

The human body's aqueous medium consists of dissolved oxygen and anions like chloride, phosphate, and bicarbonate; cations like K^+^, Na^+^, Mg^2+^, and Ca^2+^; organic compounds of low-molecular-weight species; and comparably high-molecular-weight polymeric components. Living molecules upset the balance of the implant's corrosion reactions by consuming the byproducts of anodic or cathodic reactions. Proteins' binding to metal ions and subsequent transport of the implant surface can upset the equilibrium between the surface electrons and the extra cations in the solution across the surface dual layer. It has been discovered that surface-absorbed proteins can inhibit oxygen diffusion, leading to localized, favored corrosion. By absorbing hydrogen from the environment, bacteria near the implant appear to accelerate the breakdown and alter the behavior of hydrogen created by the cathodic process [74].

Crevice corrosion, a localized attack within occluded regions or crevices of metallic components, occurs when metallic ions dissolve within confined areas, such as the contact surfaces between implant screws and bones. A slight indentation on the implant's surface can mitigate pitting corrosion and subsequent surface roughening. The film or coating's structure, density, and morphology influence the failure process. Also, the substrate's properties may considerably affect the observed level of adhesion. The potential impact on the long-term durability of bonding can be attributed to various factors related to the substrate, including its historical background, handling procedures, storage conditions, and preparation techniques, such as sputter cleaning. Metal ions and chloride ions react chemically, resulting in amalgamation and eventual deterioration, a process known as pitting corrosion [75]**.** When coupled with titanium (Ti), cobalt (Co), or zirconium (Zr), stainless steel alloy implants experience galvanic corrosion**.**

Ceramic coatings have shown promise as antimicrobial, anticorrosive, and bioactive coverings; examples include hydroxyapatite, titanium nitride, titanium oxynitride, titanium aluminum nitride, and numerous oxides (e.g., aluminum oxide, zinc oxide, tantalum oxide, silicon oxide, niobium oxide, and cerium oxide). The best defense against infection and corrosion is a coating made of hydroxyapatite [10].

The evaluation of the titanium nitride (TiN) coating on optical instruments (OIs) was conducted by van Hove et al. [52]. They coated the Ti–6Al–4V alloy with TiN, significantly improving its corrosion resistance. Thermal spraying was used by Saro et al. to apply a coating of hydroxyapatite (HA) to titanium Ti–6Al–4V and stainless steel (SS316L) surfaces. A hybrid HA-TiO_2_ coating was also used for the SS316L stainless steel foundation. Coatings significantly improved corrosion resistance, as seen by side-by-side test tube comparisons of coated and untreated samples [76]. The Ti–6Al–4V specimen, covered with a thickness of 30 μm, had the lowest recorded corrosion rate among all the coated models [77].

Additionally, corrosion was prevented by employing a niobium (Nb) intermediate layer as a barrier against seepage. The outcomes verify the efficiency and suitability of the coatings for use on surgical implements. In their research, they found that using TiAlN, especially the Nb/TiAlN double-layer coating, was significantly more effective in protecting the stainless-steel substrate from oxidation [5]. Electrochemical impedance data and potentiodynamic polarization experiment results show that a TiAlN-coated specimen has a corrosion potential of 0.128 V, significantly lower than that of an uncoated illustration made of commercially pure titanium. This work was done by Subramanian et al., who used a DC reactive magnetron sputtering method to apply coatings of TiN, TiAlN, and TiON onto a Cp-Ti substrate. Previous research has shown that the TiAlN coating has better corrosion resistance than other layers made of TiN and TiON [78].

High-speed cutting tools, aircraft components, and gas turbines frequently use nitride-based hard coatings for protection. This is because of their superior properties, such as high hardness, corrosion resistance, high-temperature stability, low friction, and the ability to protect against wear and corrosion, extending the useful life of these tools and components. By substituting a small amount of Si for Cr or Ti atoms in the coating patterns CrAlSiN and TiAlSiN, lattice distortion is induced due to the different atomic sizes. In addition, the structure of the amorphous Si_3_N_4_ matrix surrounding the crystalline phases controls the grain development of these coatings, which is what gives them their extraordinary hardness. When silicon is incorporated, the CrAlN and TiAlN ternary ceramic coatings gain improved high-temperature stability, wear resistance, and corrosion resistance [73]. Table 6 represents different types of corrosion in other parts of the human body [79].

**Table 6:** Different types of corrosion in other parts of the human body [74].

Type of Corrosion	Material	Implant Location	Shape of the Implant
Crevice	316 L stainless steel	Bone plates and screws	
Pitting	304 SS, cobalt-based alloy	Orthopedic/Dental alloy	
Corrosion fatigue	316 SS, CoCrNiFe	Bone cement	
Galvanic	304SS/316SS, CoCr + Ti–6Al–4V, 316SS/Ti–6Al–4V Or CoCrMo	Oral Implants Skrews and nuts	
Fretting	Ti–6Al–4V, CoCrSS	Ball Joints	

### 4.3 Biocompatibility of Biomedical Implant

Biocompatibility is the term used to describe the state of affairs when a biomaterial exists within a physiological environment without adversely and significantly affecting any of the other body of environment and material [80]. Biocompatibility is one of the most important factors when considering biomedical implants. These devices are surgically inserted into a person's body to replace, support, or enhance damaged or absent tissues.

**Fig. 14:**
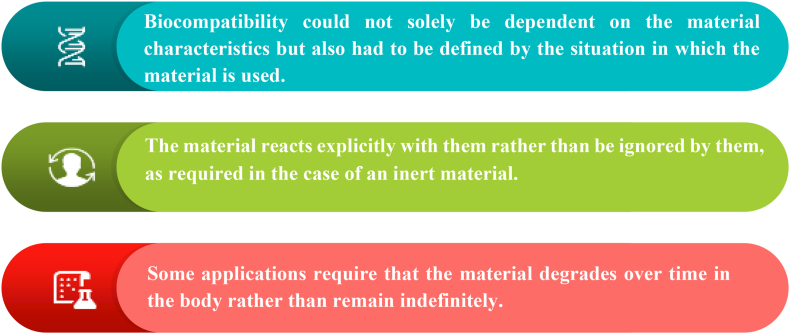
Biocompatibility of biomedical implant.

Biocompatibility is essential to lessen the restrictions posed by metallic implants. The most important thing is taking precautions, so the implant doesn't cause injury to the body (via techniques like stress shielding) or cause fibrous tissue to form and weaken the implant. This would guarantee that the device stays in the host body long enough to produce the desired biological response [81]. Fig. 14 provides a comprehensive depiction of the biocompatibility of a biomedical implant.

Coating biocompatibility was evaluated in vitro using mesenchymal stem cells isolated from mouse bone marrow. *Escherichia coli*, or *E. coli* as it is more frequently known, was used in studies on antibiotic efficacy. Fluorescence and survivability results of samples coated with 120W demonstrated their significant biocompatibility. Gene expression data was collected across the osteogenic development phase and compared to coatings placed at 20, 40, 60, and 100W power levels. Because of its better microstructural properties, the 120W layer helps modify the surface of orthopedic implants.

Due to their high strength and low affinity for physiological fluids or oxygen, titanium alloys have become the material of choice for most bone implants. In addition, it is biocompatible, has a high tensile strength, and is corrosion resistant. Titanium is used in place of stainless steel because porous AISI 316L stainless steel is prone to crevice corrosion, uniformly applying adherent layers so that handling and The bio-reactive layer inside the porous material remain effective even after surgical modification of the device [82] Several initiatives have been made to modify the chemical properties and composition of titanium surfaces, such as applying bioactive coatings to enhance the biocompatibility of titanium implants [83].

To improve the weak surface performance of substrates, transition metal nitrides like CrN and TiN coatings are frequently used as highly effective wear-resistant materials [84]. Because of their poor thermal stability and inability to adhere to cells, these materials have limited utility as bioimplants. According to the literature, using amorphous silicon may improve the biocompatibility of implants, according to the literature [85].

In Trivedi et al.'s study [86], Ti–N coatings on Ti alloys were created by magnetron sputtering, and their biocompatibility and antibacterial activity were tested. The authors of this work have developed a Ti–Si–N coating on Ti alloys to enhance the implant's cytocompatibility, cell proliferation, and differentiation. If these coatings are used, not only will their biocompatibility increase but so will their wear resistance [87]. Hydroxyapatite (HAP) has been widely used as a coating material for dental and orthopedic applications because of its high bioactivity and biocompatibility.

Implants can only be successful if they are biocompatible with the body and don't harm the cells that make them up. Titanium metals are inert. Thus, they do not stimulate the growth of bone cells or osteoblasts. The excellent mechanical qualities and positive biological activity of a medical titanium alloy (Ti–6Al–4V) can be improved by including hydroxyapatite (HAP) in the matrix. However, under physiological conditions, the HAP coating will delaminate from the substrate because of how poorly the two adhere to one another [5].

Improving biocompatibility is a significant difficulty because metallic biomaterials are inert by nature. Metal ion sensitivity, infection prevention, early-stage osseointegration, reduced stiffness, and bio-resorbability are all challenges the next generation of metallic implant materials must address in addition to improving biocompatibility [88].

### 4.4 Surface topography

Titanium was deposited in a thin layer over several biomaterial surfaces by physical vapor deposition (PVD). The PVD process did not significantly alter the surface topography of any samples. The light titanium covering completely hid the underlying chemistry of the plasma sprayed HA surface, and its chemistry was identical to that of the commercially pure titanium disks. There were no discernible differences in Ti concentration between the unmasked and masked surfaces when aliquots of the growth media were analyzed. The underlying surface chemistry was successfully masked by applying a homogeneous layer of Ti through physical vapor deposition (PVD) onto HA coatings, which also preserved the topography of the substrate. Dental implant surfaces may undergo nanoscale modification, which has the potential to alter their topography and chemistry. Increased osseointegration caused by nanoscale changes to implant surfaces has been linked to a better understanding of nanotopography's function [89]. Nanostructures affect cell behavior due to their size and density. Osseointegration and dental implant therapies may benefit from surface nanopatterning, nanocoating, and functionalization since they can dramatically increase cellular and tissue responses [90].

**Fig. 15:**
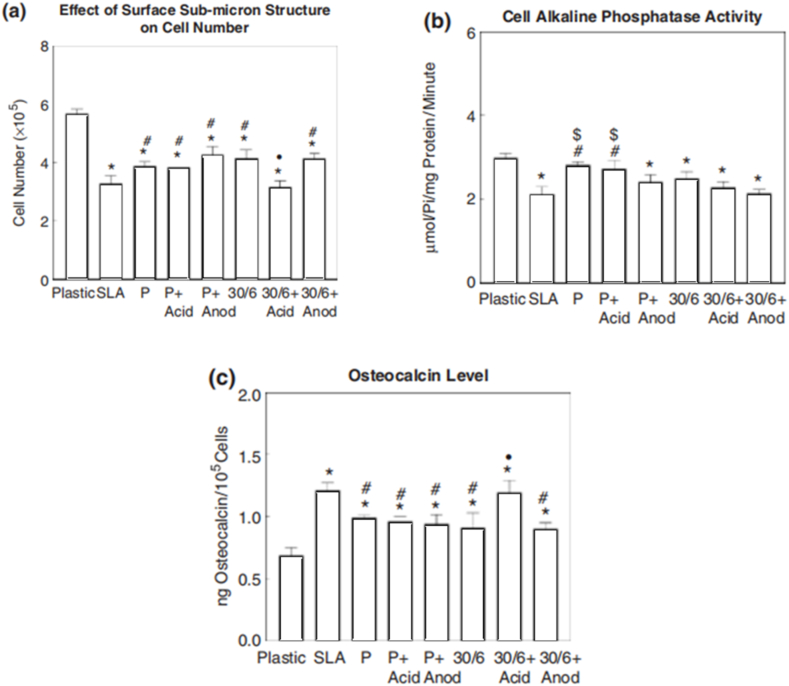
The study investigated the impact of different surface textures on MG63 cell behavior, evaluating tissue culture plastic, grit-blasted and acid-etched titanium (SLA), mechanically polished titanium (P), and electrochemically machined microstructured surfaces (30/6). Additional submicron textures were applied to polished and microstructured surfaces through acid treatment or anodization. After five days, cell numbers (a), alkaline phosphatase activity in detached cells (b), and osteocalcin levels in conditioned media (c) were assessed. ANOVA and Bonferroni-modified t-tests revealed significant differences in cell response across surface types compared to plastic, SLA, and others [91].

The impact of different surface textures on MG63 cell behavior was investigated in a study (Fig. 15(a–c)), which evaluated various surfaces including tissue culture plastic, grit-blasted and acid-etched titanium (SLA), mechanically polished titanium (P), and electrochemically machined microstructured surfaces (30/6). Additional submicron textures were applied to polished and microstructured surfaces through acid treatment or anodization. Significant differences in cell response across surface types were revealed by ANOVA and Bonferroni-modified t-tests [91].

**Fig. 16:**
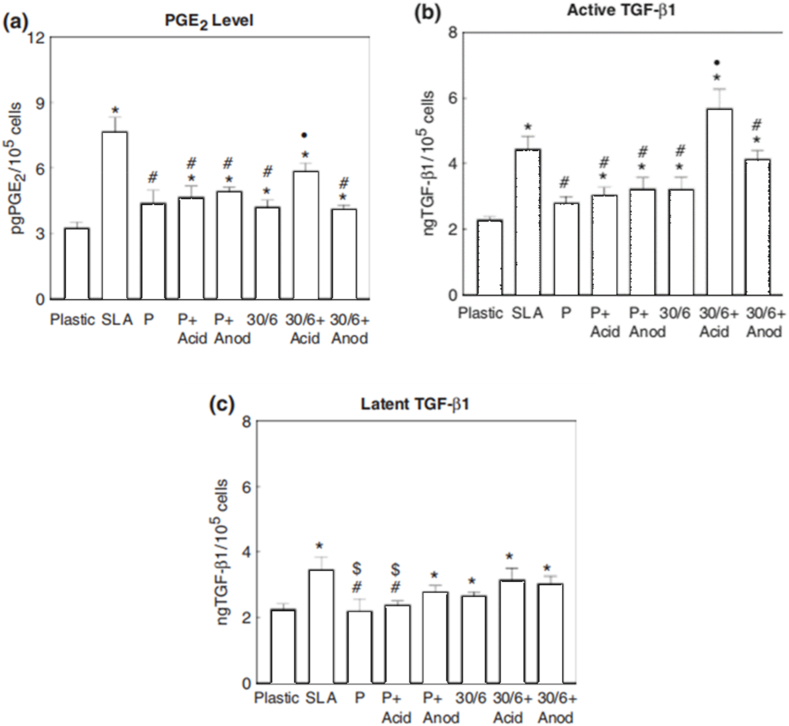
The study examined the influence of submicron surface textures on local factor levels in MG63 cell cultures. Surfaces included tissue culture plastic (plastic), grit-blasted and acid-etched titanium (SLA), mechanically polished titanium (P), and microstructured surfaces via electrochemical machining (30/6). Additional submicron textures were created through acid treatment (þ acid) or anodization (þ anod) on polished and microstructured surfaces. PGE2 levels were measured using RIA (a), active TGF-b1 levels with ELISA (b), and latent TGF-b1 levels by subtracting active TGF-b1 from acidified total TGF-b1 (c). Data represents mean ± SEM from six independent cultures. ANOVA and Bonferroni-modified t-tests indicated significant differences among surface types compared to plastic, SLA, other surfaces, and a specific condition (30/6þ acid) [91].

In another study (Fig. 16(a–c)), the influence of submicron surface textures on local factor levels in MG63 cell cultures was examined. Surfaces included tissue culture plastic (plastic), SLA, P, and microstructured surfaces via electrochemical machining (30/6). Additional submicron textures were created through acid treatment or anodization on polished and microstructured surfaces. Significant differences among surface types in PGE2 levels (measured using RIA), active TGF-b1 levels (ELISA), and latent TGF-b1 levels were indicated by ANOVA and Bonferroni-modified t-tests [91].

## 5 Coatings for interventional medical devices

Metal and plastic are the usual materials for interventional surgical tools. Tubes used in interventional medical procedures benefit significantly from being made of high-performance polymers due to these materials' lightweight, durability, low friction coefficient, and ease of production. Stents and guide wires typically make use of metallic components [92]. Applying a coating on catheters and guide wires necessitates attaining a super-hydrophilic and super-lubricating effect. This effect facilitates the smooth passage of the product through blood arteries and natural cavities, thereby reducing the likelihood of puncture and friction-induced damage. Moreover, the layers may possess supplementary characteristics such as anticoagulant, antibacterial, biodegradable, and medicine-release capabilities. Preliminary findings indicate that stainless steel surfaces are potentially enhanced with a tantalum metal coating to augment the material's biocompatibility and resistance to corrosion. Cardiovascular interventional therapy is predominantly employed in the area where it finds the highest frequency of utilization [25].

**Fig. 17:**
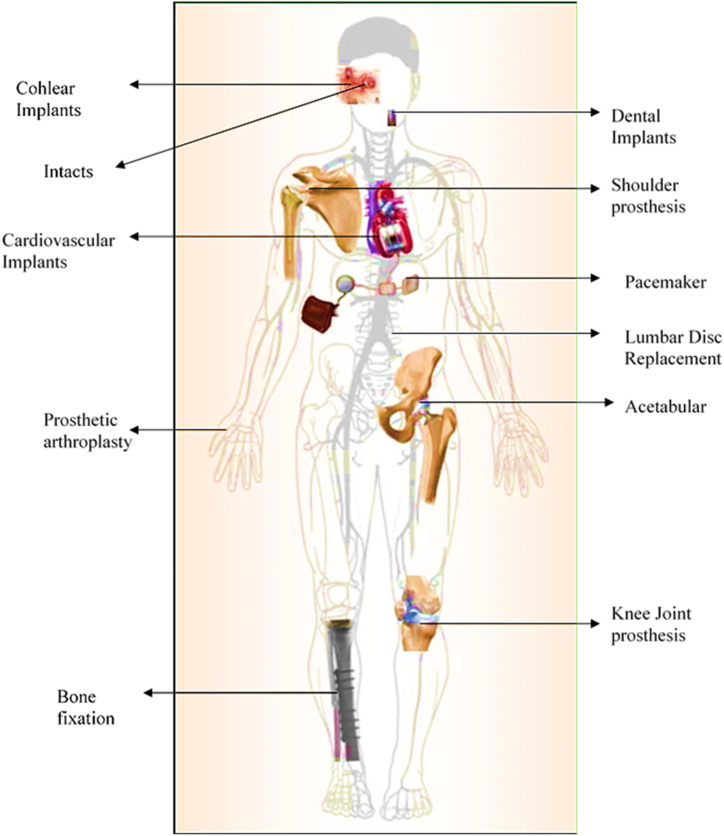
Implants on different human applications [74].

Physical vapor deposition can be used in a variety of contexts in the production of biomedical implants. Physical vapor deposition (PVD) is commonly used to deposit various substances, from metals and ceramics to polymers. The use of these materials in the manufacturing of biomedical implants is widespread. Thin material layers with improved qualities can be produced using physical vapor deposition (PVD), including increased corrosion resistance, wear, and biocompatibility. Coatings produced by physical vapor deposition (PVD) are well-suited for orthopedic implants like pacemakers and hip and knee replacements. Coatings for stents, grafts, mechanical heart valves, and other cardiovascular devices can also be produced. Fig. 17 illustrates the typical types of implants utilized in various human applications [74].

### 5.1 Orthopedic implants

Surgeons specializing in orthopedics regularly work with biomaterials like titanium, stainless steel, and CoCrMo alloys. Infections, inflammatory reactions, low corrosion resistance, variations in elastic modulus, stress shielding, and excessive wear after insertion are the most common causes of failure of orthopedic implants. As a result of the issues with implant materials, many new approaches to their design, composition, and surface qualities have been developed. It is generally agreed that coatings are an effective method of improving the functionality of implant materials.

Orthopedic applications utilize a variety of materials, including polymers, metals, alloys, ceramics, and composites [[93], [94], [95]]. These materials must be corrosion-resistant, non-toxic, biocompatible, and have outstanding physical, mechanical, and tribological qualities [96,97]. The materials most frequently utilized in orthopedic applications are presented in Table 7.

**Table 7:** Most common biomaterials for orthopedic implants [[96], [97]].

Material	Use	Advantages	Disadvantages	Challenges
Titanium Alloy	Femoral hip stem Shoulder stems Fasteners, nail, rod Facture fixation plate Pedicle screws and rods for the spine	Lightweight Less biological response Biocompatible High corrosion resistance	Poor bending ductility Poor wear resistance Expensive High modulus	Biodegradable Biological inertness Antibacterial Stability in mechanical properties Wear reduction
Cobalt Chrome Molybdenum	Bearing surface in metal Plates and wires Shorter term implants	Long term biocompatibility High corrosion resistance High wear resistance High impact durability	Stress shielding effect Poor machinability Biological toxicity due to the emission of Ni	Wear reduction Metallic fretting Biological inertness
Stainless steel alloy	plate, screws, pins, wires Sliding hip screw Cerclage cables Flexible and intramedullary nails	Widely available High ductility Accepted toughness Accepted biocompatibility	Very high modulus Allergic reactivity Stress shielding effect Poor wear resistance	Corrosion resistance Wear reduction Biological inertness
Polyethylene	Bearing surface	Biocompatibility Wear resistance	Wear debris Joint infection Lower mechanical properties	Fatigue life
Alumina/Zirconia composites	Bearing surface	high smoothness biocompatibility	High fracture rate	Brittleness

Coatings have gained popularity in improving the biological, tribological, antibacterial, and mechanical properties of implant materials used in orthopedics. Increasing biocompatibility is the primary goal of implant materials. The deposition of a substance onto a metal surface via a two-step process involving vaporization and subsequent condensation is the underlying principle of these coating technologies, outlined earlier in this text [14]. Sputtering is a typical procedure for coating metallic implant materials in orthopedics. Sputtered argon gas becomes positively charged argon vapor in an environment predominantly composed of argon. Reactive metal molecules are released when positively charged argon ions are hit with a metal substrate. These materials provide a layer of defense for metals by depositing a coating on them. A high magnetic field regulates the ionization rate in magnetron sputtering [98,99].

Therefore, before their application on orthopedic devices, it is crucial to thoroughly investigate the mechanisms by which coatings undergo delamination or deterioration. Electrochemical deposition deposits a thin, tenacious oxide, salt, or metal layer onto a substrate. The metal ion or chemical complex is electrolyzed from a solution to accomplish this.

Ti–6Al–4V and Ti–6Al–7Nb are the two most frequent titanium alloys used in medicine. Orthopedic applications use these alloys extensively because of their high resistance to corrosion, fatigue strength, impact, inherent toughness, low density, and lightweight. Despite their lack of biological activity, some substances can still harm living organisms. Therefore, enhancing the proper production of osteoblasts and new bone structures is necessary. Because of this, osteointegration could be better, the process by which implants fuse with the host's tissues. Long-term implantation leads to separating the titanium-based implant from the host tissue. Temporary fixation devices are commonly utilized in the field of orthopedics and traumatology. Implants are inserted into the fractured bones to stabilize fractures and promote bone healing. These implants should be removed once the bone has fully regained its functionality. Infection following fracture fixation is a significant issue in traumatology, with potential consequences including impaired function of the broken limb, the need for several surgeries, lengthy antibiotic treatments, and, in severe cases, amputation [100].

**Fig. 18:**
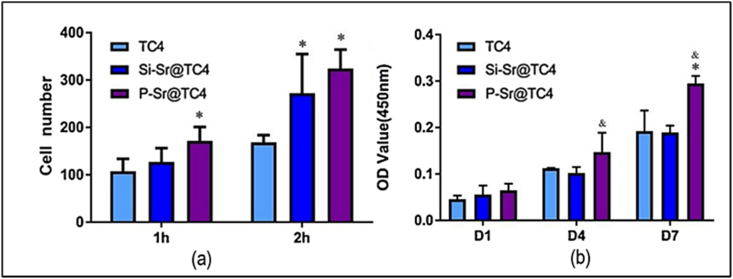
Si–Sr coating is more biocompatible than the other two coatings; (a) The number of adhered cells by nucleus fluorescent staining and counting; (b) Cell proliferation of BMSCs on different groups measured by CCK-8 assay after culture 1, 4, and 7 days [101].

Implant failure is directly related to unwanted bioactivity on the titanium surface, namely the lack of autoinduction. According to Li et al. [101], Orthopedic implants may have increased osteogenic potential by adding strontium (Sr) to calcium silicates and phosphate. Micro-arc oxidation (MAO) was used to create two coatings, calcium phosphate (P–Sr) and calcium silicate (Si–Sr), on Ti alloy, and their biological properties were compared. The results for cell adhesion and cell proliferation are shown in Fig. 18(a–b). As incubation time lengthened, more cells and higher optical density (OD) values were seen in the coated and uncoated samples. In addition to improving corrosion resistance, the data show that both coatings aid osteogenic differentiation. Titanium (TC4) substrates' potential for bone differentiation, corrosion resistance, and hydrophilicity can all be improved by the presence of both layers. Among the three coatings tested, the Si–Sr coating was the most biocompatible.

### 5.2 Antibacterial functionalization

**Fig. 19:**
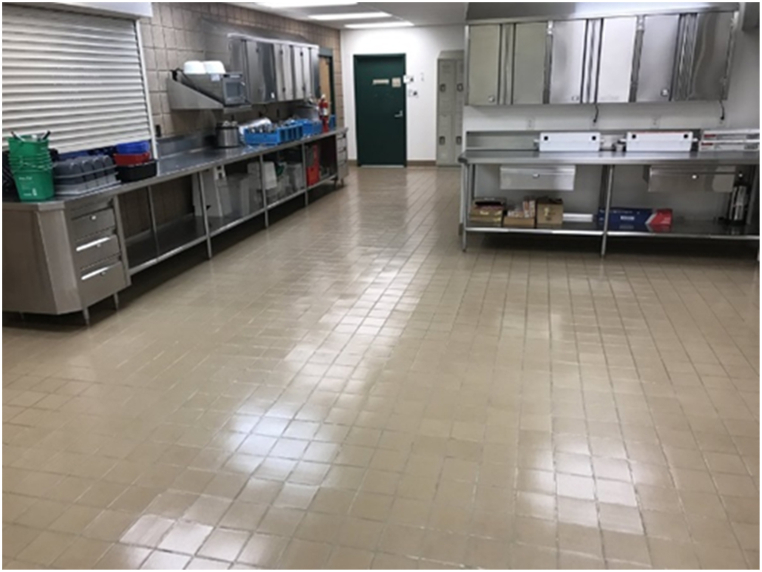
Coating on tiles for advanced flooring system [107].

In a hospital, ceramic tiles can be used for the walls and flooring of different rooms and areas. Any antimicrobial coating applied to a glazed ceramic substrate should retain the mechanical strength of the underlying ceramic [102]. There has been a significant uptick in research and development of biocide and self-cleaning surfaces since they are considered convenient options [103]. Multiple solutions are needed for different substrates, making it essential to tailor surface treatments and coatings [104]. Physical vapor deposition (PVD) coatings can be a good option in this sector. The ceramics industry has shown a keen interest in adopting novel aesthetic finishes. Physical Vapor Deposition (PVD) techniques have been widely employed to impart a metallic sheen to a wide range of tiles (Fig. 19) [105]. Titanium nitride (TiN) and chromium nitride (CrN) coatings, with silver and copper dopants, were applied to tile samples. Glow discharge was used to examine the coating's silver (Ag) and copper (Cu) dispersion. Field emission scanning electron microscopy (FE-SEM) and gamma-detected optical emission spectroscopy (GD-OES) are frequently employed in materials characterization. The deposited coatings' antibacterial efficiency was measured by the JIS Z 2801:2010 Standard test [106]. Gram-positive *S. aureus* and Gram-negative *E. coli* were the microorganisms used to evaluate silver ions' impact through the two main types of bacterial membranes.

After 24 h in a controlled medium, the number of bacterial colonies in the metal-doped TiN samples and the TiN (reference) samples was compared. A factor known as R is used in the standard to compare, on a logarithmic scale, the antibacterial surface and the number of colonies cultivated in the reference. Valid coatings were those with a bacterium mortality rate greater than 99 % (R > 2) [106].

Within 24 h of the coating being applied, all the bacteria died. When TiN + Ag was tested for its antibacterial efficacy against *S. aureus,* bacterial growth on the control TiN samples was significantly lower than expected. This result lowered the worth of resistance factors, or R factors, which are plasmids that confer antibiotic resistance on bacteria. It has been shown that titanium nitride's bacteriostatic or antibacterial activity was discovered so quickly due to levels of titanium oxide during the deposition process of the reference samples in the testing [108,109]. The coatings have a significant antibacterial impact. As the JIS Standard [77] required, the R factor was more important than two in all cases except for TiN + Ag against *S. aureus*. When metal particles are present, they produce metal ions that inhibit the growth of bacteria and other microorganisms on the surface. According to the JIS standard [110], doped physical vapor deposition (PVD) coatings offer excellent antibacterial activity while retaining the outstanding mechanical properties of undoped layers. If used in a healthcare setting, these coatings may reduce the number of bacteria that can thrive on disinfected surfaces.

**Fig. 20:**
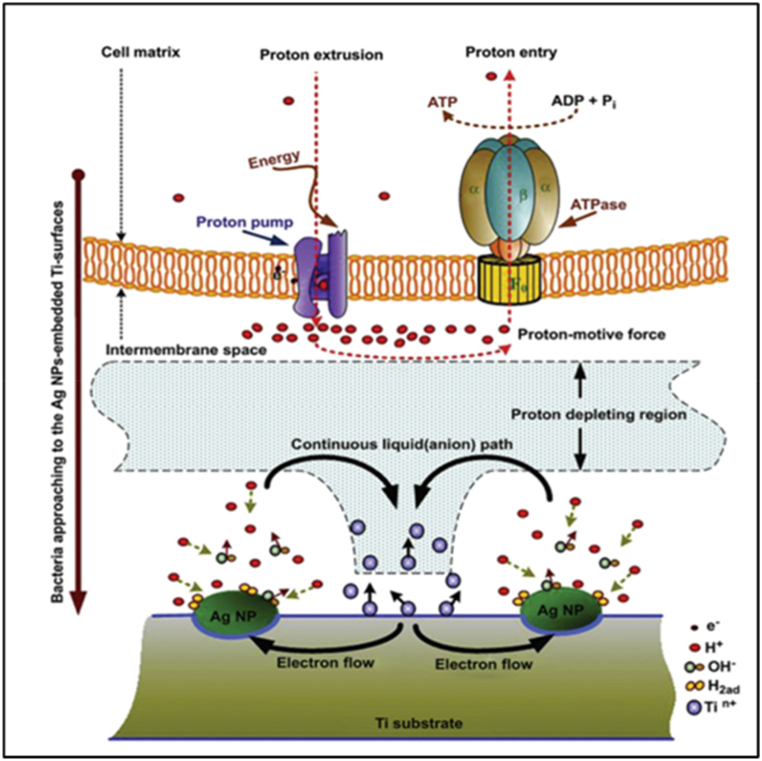
The potential antibacterial properties of surfaces impregnated with Ag nanoparticles [115].

Another method to stop bacterial adherence can be surface nanostructures. The antibacterial activity of nanoparticles depends heavily on their size and structure [111,112]. Several studies have demonstrated that Nano rough TiO_2_ nanotubes and Ti thin films effectively decrease biofilm development, adhesion, and infections. Implants with nanoscale surface patterns can help prevent oral diseases caused by *S. epidermis*, *S. aureus*, and *P. aeruginosa*. TiO_2_ nanotubes can be modified by adding silver (Ag) nanoparticles, allowing the implant to clean itself. Silver nanoparticle incorporation into various materials has demonstrated promising results in killing bacteria and viruses with little side effects on healthy cells and tissue [113]. Ag nanoparticles can induce reactive oxygen species (ROS) to aid in the inhibition of biofilm formation (Fig. 20) [113]. ROS selectively peroxidizes infectious pathogens' cell walls and membranes through a free radical chain reaction while inflicting no harm to bone, stem, or immune cells [114].

**Fig. 21:**
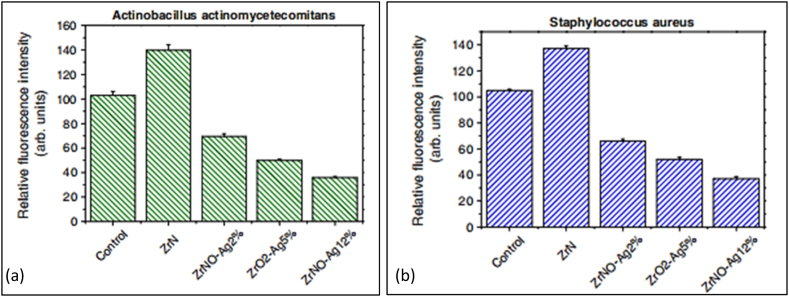
Relative fluorescence intensity of ZrN, ZrO_2_–Ag5%, ZrNO–Ag2%, and ZrNO–Ag12 % coated Ti surfaces for (a) *A. actinomycetemcomitans, and* (b) *S. aureus* colonies [118].

Developing a biocompatible and antimicrobial coating for implants is essential for increasing their success rate. Environment, bacterial characteristics, and material surface properties such as chemical composition, surface charge, hydrophobicity, and topography all play a role in the complicated process of bacterial adherence to surface roughness [116]. After 6 h of incubation, the characteristics of coated samples and pure Ti were shown to be contaminated by *A. actinomycetemcomitans* and *S. aureus*. In Fig. 21(a–b), we see the quantitative impact these bacterial cultures had on the replicas. The relative fluorescence intensity was highest for the two bacterial retentions on the ZrN. An in vitro study by B. Groessner-Schreiber et al. [108] found that ZrN coatings on titanium implants significantly decreased bacterial adherence from the mouth. Magnetron sputtering created a ZrN coating, but it did not aid in the reduction of *A. actinomycetemcomitans* and *S. aureus* in this investigation [117].

**Fig. 22:**
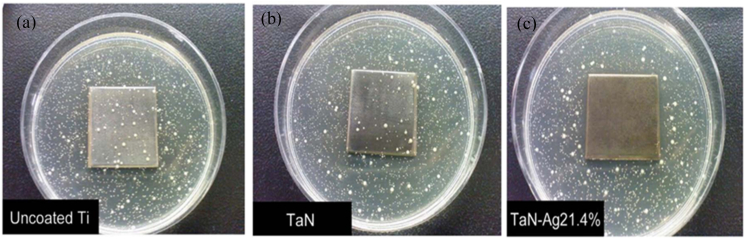
The test results to see whether bacteria could survive on uncoated, TaN, and TaN–Ag21.4 % coated Ti sample plates. S. aureus was growing, as evidenced by the colonies that could be seen on the agar plates. Each sample had bacterial growths that measured (a) 44 cfu/cm^2^ on Ti, (b) 37 cfu/cm^2^ on TaN, and (c) 3 cfu/cm^2^ on TaN–Ag [119].

The coated samples with ZrO_2_–Ag5%, ZrNO–Ag2%, and ZrNO–Ag12 % showed lower retention of *A. actomycetemcomitans* and *S. aureus* while having greater surface roughness values (Ra = 0.8–1.1 m). Short-term antibacterial efficiency was most significant for the ZrNO–Ag12 % coated Ti with the highest silver concentration (11.8 at.%), as evidenced by the lowest fluorescence intensity and, consequently, the fewest numbers of adhering bacteria. The bacterial viability test results are shown in Fig. 22(a–c). Colonies of both *Actinomyces actinomycetemcomitans* and *Staphylococcus aureus* grew on the agar plates. The lowest numbers of live *S. aureus* and *A. actinomycetemcomitans* were found in ZrNO–Ag12 % samples. Ag-based coatings on various surfaces can be antibacterial because they inhibit microbial growth, reducing the number of bacteria present [119]**.**

### 5.3 Dental implant

**Fig. 23:**
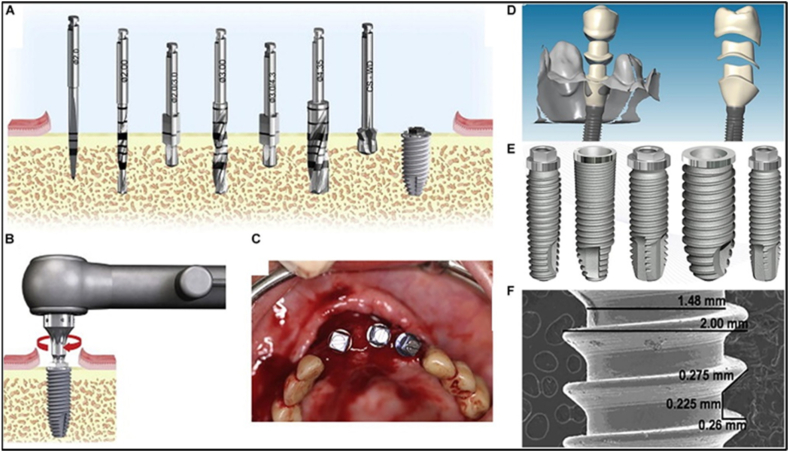
The dental implant treatment consists of the following steps: (A) deciding on the implant diameter and having it drilled to the proper depth, (B) and (C) placing the implant into the bone, and (D) attaching the prosthesis (ceramic or metal-ceramic), Dental implant examples (E, F) [123].

Dental implants are essential for replacing missing teeth due to accidents, failed root canals, agenesis, and periodontal diseases [120]. Historical methods for tooth replacement ranged from carved bamboo pegs to animal or human teeth. Modern implants, mainly made of titanium and alloys, ensure biocompatibility, corrosion resistance, mechanical strength, and osseointegration [121]. Innovations in biomaterials and nanoscale surface engineering enhance implant durability and performance, paving the way for advanced dental solutions [122]. Innovative biomaterials, advanced implant design, and nanoscale surface engineering techniques such as coating, patterning, functionalization, and molecular grafting offer promising solutions to medical challenges [120]. Fig. 23(A-F) demonstrates the sequential stages involved in dental implant treatment.

PVD coating techniques are utilized in the surface modification of dental implants, particularly at the nanoscale. These processes aim to enhance the biocompatibility and functional properties of implant surfaces. PVD coatings can alter cellular behavior by influencing protein adsorption and reducing bacterial adhesion and proliferation. They are applied to various metals and alloys used in dental implants, including titanium (Ti) and its alloys (e.g., Ti–6Al–4V). Nanostructured surfaces created through PVD have shown promising results in promoting osteoblast adhesion and proliferation, thus improving osseointegration. This technology offers potential advantages in enhancing the success and longevity of dental implants by optimizing surface topography and chemical composition at the nanoscale [124,125].

**Fig. 24:**
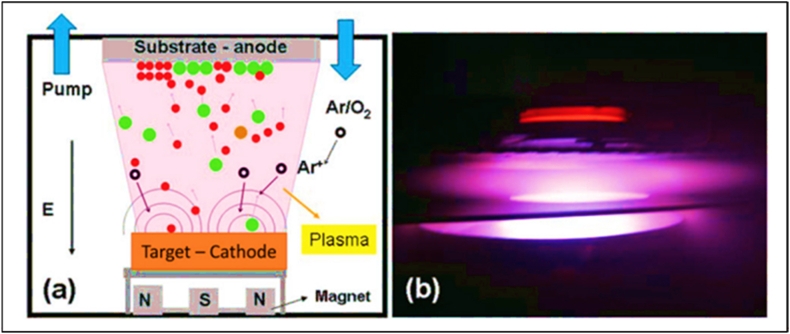
(a) A diagram illustrating the radio frequency (RF) sputtering process. (b) The arcing and deposition occur within our RF sputtering system [115].

The sputtering method is commonly used in dental implant manufacturing to apply coatings. In this method, atoms or molecules of specific materials are ejected in a vacuum chamber and deposited onto implant surfaces. This process influences material characteristics, power, sputtering time, gas flow, and operating pressure. Magnetron sputtering (MS) efficiently incorporates antimicrobial compounds into implant materials, ensuring sustained bactericidal properties. For instance, titanium dioxide (TiO_2_) coatings can be applied using sputtering methods to create micro-roughened surfaces that discourage bacterial adhesion. PVD technologies like radio frequency (RF) sputtering are also employed to deposit calcium phosphate (CaP) thin films, enhancing osseointegration potential [120]. One way to create CaP thin films on metal surfaces is by a process depicted schematically in Fig. 24(a–b) as radio frequency (RF) sputtering.

These advanced coating techniques are pivotal in preventing bacterial colonization and biofilm formation on dental implants, thereby reducing the risk of infection and improving implant success rates.

### 5.4 Cardiovascular devices

PVD coating has emerged as a revolutionary surface modification technique in cardiovascular devices [126,127]. PVD coatings have been extensively utilized to enhance the biocompatibility, mechanical properties, and overall performance of various cardiovascular implants, such as stents, guidewires, and vascular grafts. These coatings provide outstanding corrosion resistance, wear resistance, and reduced friction, thus improving the longevity and reliability of cardiovascular devices. For instance, due to their excellent biocompatibility and durability, titanium nitride (TiN) and diamond-like carbon (DLC) are commonly used in PVD coatings. TiN coating offers superior wear protection and reduced thrombogenicity, making it ideal for stents and guidewires [128].

On the other hand, DLC coatings exhibit low friction properties and biocompatibility; hence, they are often applied to vascular grafts to minimize frictional losses and enhance blood compatibility. Studies have shown that PVD-coated cardiovascular devices have significantly improved clinical outcomes by reducing the risk of restenosis, thrombosis, and inflammation. The precise control over coating thickness and composition provided by PVD technology enables tailored surface modifications to meet specific requirements of different cardiovascular applications. Consequently, PVD coating has become indispensable in advancing the performance and safety of cardiovascular devices, paving the way for innovative developments in interventional cardiology and vascular surgery [129].

## 6 Challenges of PVD coating for biomedical implants

**Fig. 25:**
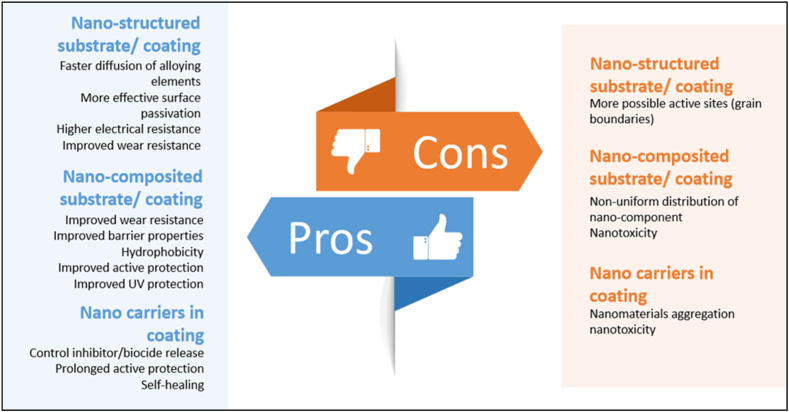
Pros and cons of PVD coating [131].

The use of expensive and complicated equipment is a significant drawback of PVD. The PVD coating industry may be more dynamic than its competitors. PVD cannot be used with substrates that have a complex geometry. Its high cost, low yield, and incessant requirement for reliable cooling system maintenance severely doubt its viability and application. Therefore, adjusting the settings to achieve optimal results is essential [130]. Adhesion, coating uniformity, contamination, process and equipment complexity, material compatibility, heat sensitivity, coating thickness restrictions, and multilayer coatings are only some of the obstacles that PVD coating must face. Scientists are investigating new coating materials for their potential advantageous properties (Fig. 25). PVD coatings may find new uses by adding these substances.

Among nanotechnology-related coatings, intelligent coatings are the most promising option. Coatings react to environmental cues (pH, humidity, coating deformation, electromagnetic radiation, etc.) by releasing corrosion inhibitors, biocides, etc., from nano reservoirs or nanocarriers scattered throughout the matrix. It has been suggested that chromate conversion coatings be phased out in favor of self-curing novel coatings. Current chromate-free pigments (silicates, borates, phosphates, molybdates, or cyanamides of Zn, Sr, Ca, Al, Ba, Mg, or Ce) do not work well in thin organic coatings with a low barrier functionality (such as coil and aircraft coatings). Water solubility and quick evaporation are not factors in selecting organic inhibitors. Some have proposed using sol-gel coatings instead of chromate conversion coatings. However, when inhibitors are introduced, they can morph into an unfavorable state. Inhibitors added to a sol-gel matrix may lose efficacy shortly [131].

Using nanotechnology in PVD coatings may have far-reaching consequences in the future. Layers with a regulated nanostructure in particle size and shape can enhance mechanical, thermal, and electrical properties. Recent research and development efforts have focused on creating alternatives to chromate conversion coatings using nanotechnology. Nanosized silica has been shown to have positive effects. Cross-linking modified silica nanoparticles made by an aqueous sol-gel procedure to generate a thin layer illustrates a nanoscience approach to coatings. This self-assembled nanophase particle surface treatment for improving topcoat layer adherence is based on hydrolyzed silanes (free of organic solvents and Cr-containing chemicals) [132].

There is current research into multifunctional coatings that combine improved wear resistance with antibacterial characteristics. These multifunctional coatings may be used in medicine, electronics, and other industries. The antimicrobial coating is a fascinating innovation in the fight against the spread of illness. Because of its low toxicity, excellent biocompatibility with human organs, high abundance, low cost, and exceptional photocatalytic potential to kill a wide range of pathogenic microbes, zinc oxide (ZnO) is one of the finest materials for antimicrobial coatings [133].

Recent progress in modeling and process control has made it possible to modify coating characteristics for specific applications. It is possible to get the required effects by adjusting the coating's thickness, composition, and structure. When plasma spray techniques create a layer, the resulting hydroxyapatite (HAP) will have a different chemical and phase composition, crystallinity, crystallite size, and defect density than natural bone apatite. However, for optimal performance in vivo, these coatings require biological qualities such as osteoconductive or osteoinductivity. However, to be biocompatible, harmful substances must be absent [134].

Sometimes, the underlying complexity of the human-natural system needs to be addressed or more concisely characterized to evaluate biological reactions to materials with widely variable properties that are ingested. However, in vitro tests designed to predict the in vivo performance of a given biomaterial frequently deliver ambiguous results, highlighting the need to capture the biological complexity of living tissue in a comprehensive in vitro model and establish tractable property-function relationships.

## 7 Challenges encountered in the context of commercial applications

Bridges and offshore platforms are just two examples of the various industrial components that benefit from high-velocity oxygen fuel (HVOF) coatings, which have earned a solid reputation over the years. It is a thick cermet coating coupled with a top nitride film that is fabricated using a combination of high-velocity oxygen fuel (HVOF) and physical vapor deposition (PVD) technologies to enhance the load-bearing capability, bonding strength and wear resistance [135]. However, there are considerable obstacles to getting a PVD layer to protect large-scale structural components. Polishing HVOF coatings to a high grade is problematic because it is necessary to make tiny precision components. Consequently, the difficulties experienced in industrial applications are the appropriate proportions and achieving a smooth surface. The availability of various duplex coatings complicates efforts to standardize the industry. HVOF and PVD coatings allow for some leeway in material choice. The HVOF-PVD duplex coating chemistry is constantly changing. The testing process can be made more effective by using simulation calculations for structural design and performance prediction of duplex coatings. Adding duplex layers will be a huge step forward [136].

In another research, Gautam et al. [137] found that several titanium alloys had drawbacks. The Ti–6Al–4V alloy was found to be toxic, the Ti–Zr alloy was found to be mechanically unstable and manufacturing complex, and the Ti–Ni alloy was found to be poisonous and to have poor heat conductivity. PIII&D, CVD, and other surface modification methods must be studied to help materials achieve high levels of biocompatibility and enhanced mechanical capabilities. Medical implant results with titanium alloys have been positive. There is, nonetheless, room for advancement in terms of lessening their nanotoxicity and streamlining their synthesis. Implantable materials have progressed to include a wide range of synthetic polymeric materials. These materials are typically used as a coating to strengthen and improve the qualities of metallic implants. Because around 70 % of human bone consists of calcium phosphate (hydroxyapatite), a polymer made from hydroxyapatite is frequently used because of the material's similarity to human bone.

Functionally graded coatings (FGCs) represent an innovative category of coatings with heterogeneous structures, distinguished by gradients in their properties. Typically, FGCs consist of multiple phases combined in a manner where the structure or composition of each phase transitions smoothly across its thickness. This design aims to mitigate thermal residual stresses effectively [138]. Moreover, after being applied to a substrate, FGCs increase the surface's hardness, toughness, resilience to environmental conditions, and adhesive characteristics. These coatings can significantly enhance the surface properties of substrates compared to coatings. Because of their superior mechanical and tribological qualities, FGCs have advanced quickly and are becoming increasingly technically sophisticated. Many fields benefit from engineering innovations, but none more so than medicine. Gradient coatings have been shown to significantly affect wear and corrosion rates, enhance microstructure, and boost mechanical properties when applied to substrate surfaces. Increased interest in creating unique gradient coatings with multiple applications is anticipated because of the findings of this critical review. It will also encourage more research and design in this exciting field [139].

Accurately predicting film properties by dissecting the resulting microstructures and employing complex characterization methods for use in the biomedical industry. The study looks into the potential of thin-film coating technology and the essentials of achieving high-quality coatings. The primary characteristics, appropriate ceramics and substrates, prospective future applications, and current state of thin film coating techniques were all evaluated briefly by M. Sathish et al. [140]. Readers are given the tools they need to make educated decisions on thin films and coating structures, thanks to the thoroughness and organization of this study. Researchers can find possible applications for high-temperature, wear, and corrosion-resistant coatings by employing coating processes with well-defined core goals. This calls for a close inspection of coating micrographs, the extrapolation of research findings, and the schematic summarization of their many uses, qualities, and application domains [120].

## 8 Prospects of PVD coating in biomedical implants

Recent advancements in technology enable simulations to be conducted using specialized software tools. Since the physical vapor deposition (PVD) process has been demonstrated to have higher energy consumption, optimizing its energy usage is one of the critical challenges facing the business. External devices have improved physical vapor deposition (PVD) methods, focusing on the HiPIMS/HPPMS technique to better coating deposition techniques [141]. Accelerated ionization rates and improved charge states of the targeted ions make this method particularly promising for coatings. Researchers have stepped up their efforts to find answers that take initiative needs into account after hearing them voiced by businesses. As technology develops, improvements in the deposition methods are expected [142].

The development of PVD coatings for medical equipment will primarily concentrate on three areas: Research into novel coating materials, refinements to biocompatibility testing procedures, and coating preparation methods are all on the docket. This aspect ensures that unique coating components will acquire more attention in forthcoming styles. For instance, magnesium ions may boost osteoblast activity, encouraging osteoblast adhesion and proliferation. Increased amounts of collagen X and vascular endothelial growth factor in bone tissue contribute to bone repair [5].

Silver ions have antimicrobial properties. By decreasing osteoclast activation and enhancing osteoblast activity, strontium ions can effectively promote osseointegration [143]. Coatings must pass strict biocompatibility tests before being applied to medical equipment. This assessment is essential since the patient's health and safety depend on the biocompatibility and quality of implanted devices. Shortly after that, numerous deposition processes are anticipated to be merged into one another during the preparation of medicinal coatings. Using vapor deposition technology, a nano-coating is applied to the substrate's surface, and then an organic substance is deposited on top of the nano-coating. Super-hydrophilicity, super-lubricity, antibacterial qualities, and drug release capabilities are some benefits added to medical devices through processes including electroplating, spraying, and sol-gel technology [25].

However, modern dental implants face a significant challenge in achieving mechanical qualities close to bone tissue. Additionally, there must be a requirement for more therapeutically effective methods of optimizing implant surface alterations. Nanotechnology has provided new opportunities for developing the next generation of bone implants by facilitating the manipulation and manufacture of implants that mimic the natural topography of bones [144]. The processing of nanomaterials is an emerging field of study. Although significant research has been conducted using nanotechnology in implant surface engineering, developing dental implants with remarkable features remains challenging. Nanotoxicity testing is crucial for establishing if nanoparticles pose risks to human health. As a result, more research must be done to develop the ideal dental implant [145].

Oxide ceramic implants come with a few caveats. One such limitation is the subpar sintering performance of chromium oxide in ambient air. Another issue with zirconia-based implants is that their quality declines dramatically as they age because their crystal structure changes from tetragonal to monoclinic. Multiple filler and reinforcing material proposals were made to deal with these problems. Hip implant material with desirable physical and mechanical qualities and higher resistance to wear was developed by carefully balancing different ceramics and employing the appropriate processing and manufacturing conditions [93].

Several physical vapor deposition processes, including cathodic arc deposition, DC reactive magnetron sputtering, and close field magnetron sputter ion plating, have shown promise in improving coatings' biocompatibility, corrosion resistance, and mechanical qualities. Hydroxyapatite (HAP), bioactive glass (BG), and titanium nitride (TiN) are just a few of the coating materials that have been shown to improve performance significantly. However, these coatings require durability, adherence, and performance degradation enhancements in an industrial context. Cathodic arc deposition, DC reactive magnetron sputtering, and close field magnetron sputter ion plating are all examples of physical vapor deposition techniques that have shown promise in enhancing coatings' biocompatibility, corrosion resistance, and mechanical qualities. However, these coatings' durability, adhesion, and degradation characteristics create significant obstacles that restrict their usage in industrial environments [94].

Since the hardness and modulus of an HVOF coating are similar to those of a soft substrate and a hard PVD coating, a smooth transition to a new shape and strong reinforcement are possible. Since the “eggshell effect” is brought on by the substrate plastically deforming under high loads due to stress concentration, an HVOF interlayer may help mitigate this phenomenon. The HVOF interlayer prevents the PVD coating from delaminating and peeling catastrophically. Instead of removing the coating during the sliding process, shearing causes it to be lost. Duplex coatings have longer lifespans than PVD coatings [136].

## 9 Conclusion

Applying a coating created through physical vapor deposition (PVD) is a flexible method that can improve various surface qualities, making them more appropriate for applications. The coating material and process parameters of PVD can increase multiple attributes, such as hardness, wear resistance, corrosion resistance, adhesion, biocompatibility, and so on. These qualities, along with many others, are amenable to improvement. This coating offers antibacterial qualities and is highly popular; the most typical application for PVD coating is on biomedical implants, including those used in orthopedics, dentistry, and cardiovascular care. This coating is utilized on biomedical implants.

PVD coating provides several benefits, including reduced wear and friction, enhanced resistance to corrosion, increased biocompatibility, improved osseointegration, and an overall more aesthetically pleasing look. Coatings made of hydroxyapatite (HAP) and diamond-like carbon (DLC) are often used on dental implants. Hydroxyapatite is used because it promotes the formation of bone as well as adhesion to the surface of the implant. Diamond-like carbon, on the other hand, is utilized due to its low friction, high wear resistance, and exceptional biocompatibility. PVD coatings can assist in lowering the risk of disease by inhibiting the growth and spread of germs, which can be accomplished by helping to impede the creation and multiplication of germs. This can be done by incorporating antibacterial substances in the coatings, which can then be applied to surfaces. These surfaces improve patient safety and general cleanliness by combining the benefits of PVD coatings with antibacterial activity. These benefits include avoiding infections, increased protection longevity and decreased reliance on antibiotics.

## Funding

The author(s) declare that no financial support has been received for this article's research, authorship, and publication.

## Data availability statement

Data used to support the findings of this study are available from the corresponding author upon request.

## CRediT authorship contribution statement

**Khondoker Safin Kaosar Saad:** Writing – original draft, Conceptualization. **Tasfia Saba:** Writing – original draft, Conceptualization. **Adib Bin Rashid:** Writing – review & editing, Methodology, Conceptualization.

## Declaration of competing interest

The authors declare that they have no known competing financial interests or personal relationships that could have appeared to influence the work reported in this paper.
